# Public trust is earned: Historical discrimination, carceral violence, and the COVID‐19 pandemic

**DOI:** 10.1111/1475-6773.14187

**Published:** 2023-06-06

**Authors:** Andrew Anderson, Demar F. Lewis, Paul Shafer, Jordan Anderson, Thomas A. LaVeist

**Affiliations:** ^1^ Health Policy & Management Tulane University New Orleans Louisiana USA; ^2^ Department of African American Studies Yale University New Haven Connecticut USA; ^3^ National Center for Chronic Disease Prevention and Health Promotion Boston University Boston Massachusetts USA; ^4^ National Center for Chronic Disease Prevention and Health Promotion Centers for Disease Control & Prevention Atlanta Georgia USA

**Keywords:** health equity, questionnaire design, social determinants of health, survey research

## Abstract

**Objective:**

To assess whether knowledge of Tuskegee, the U.S. Immigration and Customs Enforcement (ICE) agency's detainment of children, and satisfaction with the George Floyd death investigation were associated with trust in actors involved in the development and distribution of coronavirus vaccines.

**Data Sources and Study Setting:**

National survey with a convenience sample of Black (*n* = 1019) and Hispanic (*n* = 994) adults between July 1 and 26, 2021.

**Study Design:**

Observational study using stratified adjusted logistic regression models to measure the association between ratings of the trustworthiness of actors involved in the development and distribution of coronavirus vaccines.

**Principal Findings:**

Among Black respondents, lower satisfaction with the George Floyd death investigation was associated with lower trustworthiness ratings of pharmaceutical companies (ME: −0.09; CI: −0.15, 0.02), the FDA (ME: −0.07; CI: −0.14, −0.00), the Trump Administration (ME: −0.09; CI: −0.16, −0.02), the Biden Administration (ME: −0.07, CI: −0.10, 0.04), and elected officials (ME: −0.10, CI: −0.18, −0.03). Among Hispanic respondents, lower satisfaction was associated with lower trustworthiness ratings of the Trump Administration (ME: −0.14, CI: −0.22, −0.06) and elected officials (ME: −0.11; CI: −0.19, −0.02). Greater knowledge of ICE's detainment of children and families among Hispanic respondents was associated with lower trustworthiness ratings of state elected officials (ME: −0.09, CI: −0.16, 0.01). Greater knowledge of the US Public Health Service Study of Syphilis in Tuskegee was associated with higher trustworthiness ratings of their usual source of care (ME: 0.09; CI: 0.28, 0.15) among Black respondents (ME: 0.09; CI: 0.01, 0.16).

**Conclusions:**

Among Black respondents, lower satisfaction with the George Floyd death investigation was associated with lowered levels of trust in pharmaceutical companies, some government officials, and administrators; it was not associated with the erosion of trust in direct sources of health care delivery, information, or regulation. Among Hispanic respondents, greater knowledge of the ICE detainments was associated with lower trustworthiness ratings of elected state officials. Paradoxically, higher knowledge of the Study of Syphilis in Tuskegee was associated with higher trustworthiness ratings in usual sources of care.

**What is known on this topic**

- Scholars attributed low early uptake of the COVID‐19 vaccines among Black and Hispanic Americans to medical mistrust.
- Medical mistrust is associated with experiences with racism and discrimination in the health care system and beyond.
- US media and the scientific community have named perceptions of historical and contemporary atrocities as contributors to low levels of trust of medical professionals among Black and Hispanic communities.

**What this study adds**

- Black respondents who were less satisfied with the George Floyd death investigation had lower trustworthiness ratings of pharmaceutical companies and government, but not sources of health care information, regulation, or delivery (i.e., usual care source, vaccine clinics, the FDA).
- Hispanic respondents with greater knowledge of the ICE's detainment of children and families had lower trustworthiness ratings of state elected officials.
- State‐sanctioned structural violence in racially and ethnically minoritized communities is associated with racially and ethnically concordant lower levels of trust in COVID vaccine‐related actors.

## 1 INTRODUCTION

The low early uptake of coronavirus vaccines among Black and Hispanic populations in the United States has been attributed to widespread medical mistrust rather than the misconduct of government and medical institutions.^1^, ^2^, ^3^, ^4^ Group‐based knowledge of unequal treatment, neglect, and violence has led many to consider these institutions untrustworthy. Given the importance of laws and regulations in shaping health outcomes,^5^, ^6^ scholars have offered frameworks to expand analyses of institutional trust to distinguish between trust, distrust, mistrust, and trustworthiness and the important implications of each for public health and state actors.^7^, ^8^, ^9^, ^10^, ^11^, ^12^, ^13^, ^14^, ^15^ Public perception of the trustworthiness (i.e., the perceived reliability of institutional actors to perform their duties or publicly stated commitments) of actors involved in the COVID‐19 pandemic response predates the pandemic. The reputations of these actors may have improved or worsened as a result of actions taken to address the pandemic. Yet, less is known about whether knowledge of historical *and* contemporary abuses, crimes, and discrimination perpetrated by these actors influences their trustworthiness among Black and Hispanic populations.

Discrimination experienced within the health care system is a known contributor to institutional mistrust.^16^ For instance, despite mixed evidence, the US Public Health Service Study of Syphilis in Tuskegee has been repeatedly cited as a source of mistrust, particularly among Black Americans.^17^, ^18^, ^19^, ^20^, ^21^, ^22^, ^23^, ^24^ The United States has perpetrated or been complicit in health violence against Black, Hispanic, and Indigenous populations domestically and abroad (e.g., the sterilization of Native American women and girls through the Indian Health Service; the infection of prisoners, psychiatric patients, and sex workers to test the efficacy of penicillin in Guatemala), which led many to avoid health interventions proposed by actors perceived to act in partnership with those agencies and institutions.^25^, ^26^, ^27^, ^28^, ^29^ Although knowledge of historical atrocities and common experiences of racial discrimination is known to have tangible health consequences,^30^, ^31^ this domain of knowledge has important behavioral consequences for public health actors that were underexplored during the COVID‐19 pandemic.

Public health researchers and scholars across multiple disciplines have found police violence—disproportionately against Black Americans—has myriad health consequences in need of intervention.^32^, ^33^, ^34^, ^35^, ^36^, ^37^, ^38^ Research shows that indirect police contact exacerbates health risks for people living in highly surveilled neighborhoods,^39^, ^40^, ^41^, ^42^, and the effects of this police contact have varied by gender.^39^, ^43^, ^44^, ^45^ Scholars have shown that routine exposure to police violence is linked to medical mistrust,^46^ lower utilization of important health services,^39^, ^47^ and exacerbation of other known health disparities for youth and adults.^40^, ^42^, ^43^, ^48^, ^49^ Sociolegal scholars have also begun examining how legal standards are being enforced to deter police misconduct,^50^, ^51^, ^52^, ^53^, ^54^, ^55^, ^56^, ^57^ and others are beginning to ask questions about how to use available 21st‐century technological innovations to regulate law enforcement behavior more expansively.^58^, ^59^, ^60^, ^61^, ^62^, ^63^, ^64^ However, research examining the impact of police violence—or (dis)satisfaction with governmental responses to police violence—on the political behaviors of American citizens and residents remains scant, despite evidence demonstrating that knowledge of this violence also has important political consequences (particularly among racially minoritized populations).^65^, ^66^, ^67^

In this paper, we use a novel survey fielded during the summer of 2021 to empirically examine whether knowledge of historical atrocities and (dis)satisfaction with governmental responses to police violence influence trust in key actors involved with the development and distribution of the coronavirus vaccines. We tested three hypotheses to evaluate whether (1) greater dissatisfaction with governmental responses to the death of George Floyd, (2) greater knowledge of the US Public Health Service Study of Syphilis in Tuskegee, and (3) greater knowledge of the unsanitary detainment of immigrant children and families by the US Immigration and Customs Enforcement (ICE) agency were associated with lower perceptions of the trustworthiness of the developers and distributors of the coronavirus vaccines among Black and Hispanic respondents. Based on our analysis, we offer observations about the importance of intentionally reconciling historical and contemporary harms committed against Black and Hispanic populations to promote a culture of health in the United States.

## 2 METHODS

### 2.1 Study design

We fielded a national online survey (COVID‐19 Pandemic Trust, Racism, and State Violence Study) with a convenience sample of Black and Hispanic adults aged 18 and older between July 1 and July 26, 2021, using the Qualtrics survey platform. The data were scrubbed for duplicates, speeders (e.g., inappropriate completion time), flatliners (e.g., same response to all options), inattention (e.g., nonsensical responses), bad verbatim responses, and location outside of the U.S., ultimately yielding an achieved response rate of 46.6%. All respondents who completed the survey were provided a financial incentive of $6.25 on average by Qualtrics commensurate with the length of the survey (30 min). Our study protocol and survey instrument were approved by the Tulane University Social‐Behavioral Institutional Review Board.

### 2.2 Instrument and sample

Our instrument included adapted measures from the Uncovering COVID‐19 Experiences and Realities (UnCOVER) Study fielded by the Society, Health, and Racial Equity Lab at Tulane University from May to July 2020, as well as the COVID Collaborative Study fielded by Langer Research Associates.^68^, ^69^ Since the COVID Collaborative study explicitly focused on Black and Hispanic respondents, we extend this work by restricting our final analytical sample to 1911 self‐identifying Black (*n* = 1019) and Hispanic (*n* = 994) respondents (see Table 1) with complete responses for our primary outcome variables, predictor variables, and covariates.

**TABLE 1:** Sociodemographic characteristics by race and ethnicity (*n* = 1911).

	Black		Hispanic	
	*n* = 1019		*n* = 994	
Mean age	40		37	
	%	No.	%	No.
Gender				
Man	36.9	375	32.7	323
Woman	62.6	636	65.5	647
Transgender	0.5	5	1.8	18
Income				
<$24,999	43.3	358	31.9	253
$25,000–49,999	31.1	257	27.7	220
$50,000–99,999	10.3	85	17.9	142
$100,000–199,000	13.1	108	20.9	166
$200,000+	2.3	19	1.6	13
Insurance				
Private	29.6	302	36.3	361
Medicaid	24.1	246	17.9	178
Medicare	22.4	228	18.4	183
Other	16.3	166	14.1	140
Uninsured	7.6	77	13.3	132
Education				
<High school	7.1	72	5.9	59
High School/GED	25.0	255	25.1	249
Some college, no degree	25.9	264	23	229
College degree or more	42	428	46	457
Physical health status				
Excellent/very good	45.9	468	47.6	473
Good	34	346	33.2	330
Fair/poor	20.1	205	19.2	191
Mental health status				
Excellent/very good	50.1	511	45.8	455
Good	27.9	284	25.2	250
Fair/poor	22	224	29.1	289
US born				
Born outside the US	3.8	39	21.7	216
Born in US	96.2	980	78.3	778
Political affiliation				
Republican	7	71	16.8	167
Democrat	64.1	653	49.3	490
Independent	22.1	225	23.8	237
Something else	6.9	70	10.1	100
My destiny is tied to the destiny of other people of my racial and ethnic group in the US				
Strongly agree/agree	54	550	49.8	495
Neither agree nor disagree	27.9	284	30.9	307
Disagree/strongly disagree	18.2	185	19.3	192

*Note*: Summary statistics of respondents to a national survey administered between July 1 and July 26, 2021, which asked participants to rate the trustworthiness of actors involved in the development and dissemination of the coronavirus vaccines, knowledge of historical medical‐related crimes against humanity, and satisfaction with high‐profile cases of carceral violence.

[Correction added on 27 June 2023, after first online publication: the ‘Mean age’ heading has been aligned with the mean ages of 40 and 37.]

### 2.3 Measures

The outcome variables were the respondents' perceptions of the trustworthiness of actors involved in the development and distribution of the coronavirus vaccines, based on the survey items fielded during the COVID Collaborative Study.^69^ Respondents were asked, “How trustworthy are each of these actors which at various points have been involved in the development and distribution of the coronavirus vaccine?” This question was answered using a five‐point Likert scale (completely, mostly, somewhat, not much, and not at all), evaluating the following actors: (1) “Drug companies working to create and test the vaccine”, (2) “The U.S. Food and Drug Administration (FDA)”, (3) “The Trump/Pence Administration”, (4) “The Biden/Harris Administration”, (5) “Your usual doctor or health care team (skip if you don't have one)”, (6) “Pharmacies and walk‐in clinics where people can get vaccinated”, and (7) “Elected officials in your state”. We categorized participant ratings of the trustworthiness of each actor as (a) completely, (b) mostly, (c) somewhat, or (d) not much/not at all. Higher values indicate greater within‐group perceptions of the trustworthiness of these actors.

We had three key predictor variables of interest (see Table 2). The first measured participants' working knowledge of the US Public Health Service Study of Syphilis in Tuskegee. Respondents were asked, “How much, if anything, have you heard or read about the United States Federal Government's Tuskegee Syphilis Study from 1932 to 1972, in which a group of Black men in Alabama who had syphilis was not told about it or treated for it?” using a five‐category ordinal variable with Likert response options: a great deal, a lot, a moderate amount, a little, and none. Higher values indicate higher levels of knowledge about the study. Response options were recoded to delineate between respondents who knew a great deal/a lot/a moderate amount and those who knew little/none (reference group). The literature is mixed on its assessment of the association between knowledge of the Tuskegee study and levels of trust and mistrust in medical institutions.^23^, ^24^ We sought to assess whether knowledge of the study correlates with perceptions of trustworthiness, given the resurgence of the study in popular media as a reported cause of low trust leading to low early uptake of the coronavirus vaccines.

**TABLE 2:** Trust in actors involved in the COVID‐19 vaccine development by race and ethnicity.

	Black		Hispanic	
	*n* = 1019		*n* = 994	
	%	No.	%	No.
Pharmaceutical companies				
Not much/not at all	19.4	198	16.1	160
Completely/mostly/somewhat	80.6	821	83.9	834
Food & Drug Administration				
Not much/not at all	18.5	189	14.9	148
Completely/mostly/somewhat	81.5	830	85.1	846
Trump Administration				
Not much/not at all	66.8	681	55.4	551
Completely/mostly/somewhat	33.2	338	44.6	443
Biden Administration				
Not much/not at all	20.4	208	23.7	236
Completely/mostly/somewhat	79.6	811	76.3	758
Doctor or health care team				
Not much/not at all	16	163	15.3	152
Completely/mostly/somewhat	84	856	84.7	842
Vaccine clinic				
Not much/not at all	15.9	162	14.4	143
Completely/mostly/somewhat	84.1	857	85.6	851
State and local officials				
Not much/not at all	33.8	344	33.2	330
Completely/mostly/somewhat	66.2	675	66.8	664

*Note*: Summary statistics of respondents to a national survey administered between July 1 and July 26, 2021, which asked participants to rate the trustworthiness of actors involved in the development and dissemination of the coronavirus vaccines, knowledge of historical medical‐related crimes against humanity, and satisfaction with high‐profile cases of carceral violence.

Second, participants were asked about their working knowledge of the detainment practices of ICE. Respondents were asked “How much, if anything, have you heard or read about the detainment of immigrant children and families in unsanitary conditions and overcrowded facilities operated by the U.S. Immigration and Customs Enforcement?” using a five‐point Likert scale (a great deal, a lot, a moderate amount, a little, and none). Higher values indicate higher levels of knowledge about ICE's contemporary (and historical) detainment practices of children and families. The response options for this scale were restructured into new categories to delineate between respondents who knew a great deal/a lot/ a moderate amount, and a little/none (reference group).

Lastly, participants were asked a two‐part question assessing their knowledge of the circumstances that led to the death of George Floyd (a high‐profile death of an unarmed Black man by a police officer during the pandemic) and their level of satisfaction with the governmental response to his death. We chose satisfaction with the outcomes of the George Floyd death investigation as a proxy for attitudes towards carceral violence during the pandemic because it was the highest profile case (garnering worldwide attention). In addition, Derek Chauvin, the officer judged primarily responsible for George Floyd's death, was convicted of his death on June 25, 2021, which was less than a week before our survey entered the field. Respondents were first asked, “How much, if anything, have you read or heard of the circumstances of George Floyd's death in Minneapolis, Minnesota on May 25, 2020?” using a five‐category Likert scale (a great deal, a lot, a moderate amount, a little, and none). Subsequently, respondents selecting response options 1–4 were asked a follow‐up question, “How satisfied are you with how Minnesota state and local government officials handled the investigation into his death?” on a different five‐point Likert scale (extremely satisfied, somewhat satisfied, neither satisfied nor dissatisfied, somewhat dissatisfied, and extremely dissatisfied). Higher values on the scale indicated higher levels of satisfaction with government responses, predicated on knowing at least “a little” about the case. We recoded this measure as a three‐item scale: extremely/somewhat satisfied (reference group), neither satisfied nor dissatisfied, and somewhat/extremely dissatisfied based on the distribution of responses and to ease interpretation.

We measured several covariates based on existing literature describing the relationships between trust and health service use. Our covariates included race/ethnicity, sex, age, household income, health insurance status, highest level of educational attainment, physical and mental health status, whether they were born in the United States, the extent to which respondents viewed their destiny in life as linked to other members of their racial/ethnic group (i.e., linked fate), and political affiliation. We also include “linked fate” in this analysis to account for known predictors of carceral knowledge and trust in state actors from literature in political science.^65^, ^70^ The self‐identified race and ethnicity of the participant were measured using a specific procedure. First, participants were asked if they identified as “Hispanic/Latinx” using a binary indicator. Next, participants were asked to select which racial group(s) they identify with (e.g., American Indian/Native American/Alaska Native, Asian, Black/African American, Native Hawaiian or Pacific Islander, or White), and participants who selected “American Indian/Native American/Alaska Native” were also given the opportunity to list their tribal affiliation(s). Finally, we asked participants to select which race and/or ethnicity they most closely identify with (e.g., Black/African American, Hispanic/Latinx, Biracial/Multiracial) to inform our analytical categorization of individual participants who may have selected one or more racial/ethnic identities. Physical and mental self‐reported health status was assessed by asking participants whether, in general, they would rate their physical and mental health as poor, fair, good, very good, or excellent.^71^ Respondents were asked to estimate their annual household income as <$24,999, $25,000–49,999, $50,000–99,999, $100,000–199,000, or $200,000 or more. Respondents reported their insurance status as having employer‐sponsored insurance (their own or that of a family member) (i.e., private), Medicaid (including Medicaid HMO/CMO), Medicare, other public insurance (e.g., Military Health Care or VA, Indian Health Service, and COBRA), or being uninsured. Participants were asked about their highest level of educational attainment, and their responses were categorized as less than high school, high school or GED, some colleges without a degree, or an associate degree or higher. Participants were also asked about their political affiliation (i.e., Republican, Democrat, Independent, or something else) and whether they were born inside or outside of the United States. The full survey instrument is included in the supplemental appendix B.

### 2.4 Analytic approach

We calculated summary statistics to assess the distribution of within‐group responses for our outcomes, exposures, and covariates (Tables 1, 2, 3). Rather than test these hypotheses by comparing relative differences in means between racial and ethnic groups (e.g., Black vs. White respondents), we explored these associations among Black and Hispanic respondents with stratified adjusted logistic regression models. Initially, we ran our analyses using the raw data from the five‐point Likert scale as categorical variables; however, we ultimately analyzed these variables as dichotomous indicators to ease the analytical interpretation of our results (the results remained the same). We report the associations using marginal effects.^72^ All data cleaning was conducted using R Studio Version 1.4.1106,^73^ and statistical analyses were conducted in Stata version 16.1.^74^

**TABLE 3:** Exposures by race and ethnicity.

	Black		Hispanic	
	*n* = 1019		*n* = 994	
	%	No.	%	No.
Knowledge of the Death of George Floyd				
Great deal/a lot/moderate	89.8	915	86.4	859
Little/none at all	10.2	104	13.6	135
Knowledge of Tuskegee Study of Untreated Syphilis				
Great deal/a lot/moderate	63	642	41.5	413
Little/none at all	37	377	58.5	581
Knowledge of U.S. Immigration and Customs Enforcement				
Great deal/a lot/moderate	69.3	706	71.1	707
Little/none at all	30.7	313	28.9	287
Satisfaction with George Floyd Investigation				
Satisfied	49.4	481	42.6	399
Neutral	20	195	26.5	248
Dissatisfied (ref)	30.6	298	30.9	290

*Note*: Summary statistics of respondents to a national survey administered between July 1 and July 26, 2021, which asked participants to rate the trustworthiness of actors involved in the development and dissemination of the coronavirus vaccines, knowledge of historical medical‐related crimes against humanity, and satisfaction with high‐profile cases of carceral violence.

## 3 RESULTS

Our sample (Table 1) included a greater proportion of participants who identified as women among Black (63.6% vs. 36.9%) and Hispanic (67.1% vs. 32.9%) respondents. Most participants were born in the United States. Nearly half of Black (43.3%) and nearly a third of Hispanic (31.9%) participants reported household incomes less than or equal to $25,000 per year. Over half of Black (53%) and nearly half of Hispanic (49.8%) participants strongly agreed/agreed with the statement that their destiny is tied to the destiny of other people of their racial and ethnic group in the US.

Most Black participants in our sample rated pharmaceutical companies (80.6%), the FDA (81.5%), the Biden Administration (79.6%), their usual doctor or health care team (84.0%), and pharmacies and walk‐in clinics where people can get vaccinated (84.1%) as trustworthy (i.e., completely/mostly/somewhat trustworthy) (Table 2). These same participants rated the elected officials in their state (66.2%) and the Trump Administration (33.2%) as not trustworthy (i.e., not much/not at all trustworthy). A similar pattern was observed among Hispanic participants. Most rated pharmaceutical companies (83.9%), the FDA (85.1%), the Biden Administration (76.3%), their doctor or health care team (84.7%), and vaccine clinics (85.6%) where they could obtain coronavirus vaccines as trustworthy. However, 55.4% of Hispanic participants rated the Trump Administration and 66.8% rated their state elected officials as not trustworthy.

Most Black (89.8%) and Hispanic (86.4%) participants had heard or read a lot about the death of George Floyd (Table 3). Most Black (63%) and nearly half of Hispanic (41.5%) respondents reported knowing a great deal/a lot/moderate amount about the Tuskegee Syphilis Study. Similarly, most Black (69.3%) and Hispanic (71.1%) participants reported knowing a great deal/a lot/a moderate amount about ICE's detainment of immigrant children and families. Overall, approximately half of Black respondents (50.6%) and 42.6% of Hispanic respondents reported not being satisfied with the outcome of the investigation into George Floyd's death.

Higher levels of knowledge of the US Public Health Service Study of Syphilis in Tuskegee were associated with rating doctors and health care teams as trustworthy (ME: 0.07; CI: 0.01, 0.13) among Black respondents (Table 4). Among Hispanic participants, higher levels of knowledge of ICE's detainment of children and families were associated with rating state elected officials as untrustworthy (ME: −0.09; CI: −0.16, 0.01), while higher levels of knowledge of the US Public Health Service Study of Syphilis in Tuskegee among these participants were associated with rating the Trump Administration as trustworthy (ME: 0.09; CI: 0.01, 0.16). We found no association between knowledge of the circumstances of George Floyd's death and ratings of trustworthiness among Black and Hispanic respondents.

**TABLE 4:** Association between trust and knowledge of high‐profile carceral violence and historical atrocities.

	Pharma		FDA		Trump/Pence		Biden/Harris		Usual Source of care		Vaccine Clinics		State Elected Officials	
	ME	CI	ME	CI	ME	CI	ME	CI	ME	CI	ME	CI	ME	CI
Black														
Knowledge of the Death of George Floyd														
Great deal/a lot/moderate	0.06	[−0.035, 0.15]	0.07	[−0.019, 0.17]	0.04	[−0.063, 0.14]	0.05	[−0.035, 0.14]	0.13**	[0.033, 0.22]	0.05	[−0.033, 0.14]	0.07	[−0.038, 0.19]
Little/none (ref)														
Knowledge of US Public Health Service Study of Syphilis in Tuskegee														
Great deal/a lot/moderate	0.04	[−0.022, 0.11]	0.01	[−0.057, 0.070]	0.05	[−0.019, 0.13]	0.06	[−0.0081, 0.12]	0.09**	[0.028, 0.15]	0.03	[−0.032, 0.089]	0.07	[−0.011, 0.14]
Little/none at all (ref)														
Knowledge of U.S. Immigration and Customs Enforcement														
Great deal/a lot/moderate	−0.05	[−0.11, 0.016]	0	[−0.067, 0.060]	−0.05	[−0.12, 0.032]	0	[−0.064, 0.060]	0.02	[−0.038, 0.081]	−0.01	[−0.071, 0.048]	−0.04	[−0.12, 0.034]
Little/none at all (ref)														
Hispanic														
Knowledge of the Death of George Floyd														
Great deal/a lot/moderate	0.04	[−0.041, 0.12]	0.05	[−0.027, 0.14]	−0.02	[−0.12, 0.075]	0.04	[−0.041, 0.13]	0.08	[−0.0065, 0.16]	0.05	[−0.036, 0.13]	0.09	[−0.014, 0.19]
Little/none at all (ref)														
Knowledge of US Public Health Service Study of Syphilis in Tuskegee														
Great deal/a lot/moderate	0.01	[−0.044, 0.070]	0	[−0.050, 0.060]	0.07*	[0.0032, 0.15]	0.03	[−0.028, 0.097]	−0.03	[−0.088, 0.029]	−0.03	[−0.084, 0.029]	0.04	[−0.035, 0.11]
Little/none at all (ref)														
Knowledge of U.S. Immigration and Customs Enforcement														
Great deal/a lot/moderate	0.02	[−0.05, 0.08]	0	[−0.06, 0.05]	−0.07	[−0.15, 0.00]	0.05	[−0.01, 0.12]	0.03	[−0.04, 0.09]	−0.02	[−0.08, 0.04]	−0.08*	[−0.15, −0.01]
Little/none at all (ref)														

*Note*: ME, average marginal effects; *CI*, 95% confidence interval; Stars indicate significance at <0.05*, <0.01**, <0.001***.

*Note*: The table reflects national from a convenience sample of people who self‐identify as Black and Hispanic. All models were adjusted for respondent age, sex assigned a birth, education, income, whether they were born in or outside of the US, political affiliation, self‐reported physical and mental health status, linked destiny, and where they consume their main source of news and information about the pandemic. Full regression results are included in a supplemental appendix.

Table 5 shifts the analytic focus from “knowledge” of the circumstances of George Floyd's death to “satisfaction” with the government's investigation of George Floyd's death. Among Black participants, on average, greater dissatisfaction in the Minnesota government's response to George Floyd's death by Derek Chauvin was associated with lower levels of trust in pharmaceutical companies (ME: −0.09; CI: −0.15, 0.02), the FDA (ME: −0.07; CI: −0.14, −0.00), the Trump Administration (ME: −0.09; CI: −0.16, −0.02), and elected officials in their state (ME: −0.10, CI: −0.18, −0.03). Similarly, lower levels of satisfaction in the investigation of George Floyd's death among Hispanic respondents were associated with lower trust in the Trump Administration (ME: −0.14, CI: −0.22, −0.06) and elected officials in their state (ME: −0.11; CI: −0.19, −0.02).

**TABLE 5:** Association between trust and satisfaction with the investigation of George Floyd's Death.

	Pharma		FDA		Trump/Pence		Biden/Harris		Usual source of care		Vaccine		State elected officials	
	ME	CI	ME	CI	ME	CI	ME	CI	ME	CI	ME	CI	ME	CI
**Black**														
Satisfaction with George Floyd Investigation (reference = satisfied)														
Dissatisfied	−0.09**	[−0.15, −0.02]	−0.07*	[−0.14, −0.01]	−0.09**	[−0.16, −0.02]	−0.08*	[−0.14, −0.01]	−0.07*	[−0.13, −0.01]	−0.03	[−0.09, 0.03]	−0.11**	[−0.18, −0.03]
Neutral	−0.07	[−0.14, 0.00]	−0.04	[−0.11, 0.03]	0.11*	[0.019, 0.20]	−0.04	[−0.11, 0.03]	−0.04	[−0.11, 0.02]	−0.03	[−0.10, 0.03]	−0.01	[−0.098, 0.07]
Satisfied (ref)														
Hispanic														
Satisfaction with George Floyd Investigation														
Dissatisfied	−0.05	[−0.12, 0.02]	−0.03	[−0.095, 0.03]	−0.15***	[−0.23, −0.07]	−0.02	[−0.089, 0.05]	0.03	[−0.04, 0.09]	0.01	[−0.05, 0.07]	−0.11**	[−0.20, −0.03]
Neutral	−0.03	[−0.09, 0.04]	−0.03	[−0.09, 0.03]	0.05	[−0.04, 0.14]	−0.08*	[−0.15, −0.00]	0.03	[−0.03, 0.10]	−0.05	[−0.11, 0.02]	0	[−0.08, 0.08]
Satisfied (ref)														

*Note*: *ME*, *Average Marginal Effects*; *CI*, 95% confidence interval; Stars indicate significance at <0.05*, <0.01**, <0.001***.

*Note*: The table reflects national from a convenience sample of people who self‐identify as Black and Hispanic. Each model was adjusted for respondent age, sex assigned a birth, education, income, whether they were born in or outside of the US, political affiliation, self‐reported physical and mental health status, linked destiny, and where they consume their main source of news and information about the pandemic. Full regression results are included in a supplemental appendix.

## 4 DISCUSSION

The US Public Health Service Study of Syphilis in Tuskegee is often cited as a signifier of historical medical racism and for the commonplace exploitation and mistreatment of Black Americans by medical institutions. Often, the study is invoked as an event that has undermined the trustworthiness of US medical institutions as well as countless other events that routinely occur during health care encounters.^75^ Despite some studies that have reported that Black Americans tend to know very little about the study, most respondents in our study reported having heard or read a moderate to great deal about it. Our finding may be due to a resurgence of news coverage describing the study during the period the survey was administered and even before the pandemic.^24^, ^76^, ^77^ In addition, previous studies have been underpowered or limited to individual cities or research settings. Our study attempted to address some of these shortcomings by recruiting a large national sample of participants. Paradoxically, we found greater knowledge of the US Public Health Service Study of Syphilis in Tuskegee was associated with higher ratings of trustworthiness for usual care teams with similar directions of association for other actors (though not statistically significant). This may be because measures of high SES (e.g., education) were associated with increased trustworthiness of actors (Supplemental Appendix B); more educated patients may be more aware of the Tuskegee study and more trusting of their usual care teams and other actors. That is, the association between trustworthiness and knowledge of the Tuskegee study may be correlational and not causal.

We also measured knowledge of the detainment of immigrant children and families in unsanitary conditions and overcrowded facilities operated by the U.S. Immigration and Customs Enforcement (ICE) agency. The ICE has maintained a detained population of 45,000 people across 131 of its listed facilities since 2003.^78^, ^79^, ^80^ The detainments drew national attention in June 2019, when Customs and Border Patrol was holding more than 15,000 detainees in crowded holding cells above their capacity (4000).^81^ Therefore, it is plausible that the actions of ICE are associated with lower ratings of trustworthiness of actors involved in the development and distribution of the coronavirus vaccines among Hispanic respondents (Hypothesis 3). We indeed found, among Hispanic respondents, that greater knowledge of these detainments was associated with lower trustworthiness ratings of state and local elected officials. State and local governments are key partners in the coordination and enforcement of federal immigration policy, and Hispanic ethnic groups from Mexico, Guatemala, and Honduras are disproportionally detained and deported.^82^, ^83^

George Floyd's death led to one of the largest social movements in US history and brought unprecedented international attention to police brutality (e.g., excessive force, false imprisonment, wrongful search and seizure). His death further challenged the trustworthiness of the people empowered by the state to ensure the safety of American citizens—the police—which may have increased suspicion of all agents of the state working to protect the health and safety of people living in the U.S. We tested whether greater dissatisfaction with the response to the death of George Floyd was associated with lower trustworthiness ratings. We found most Black and Hispanic respondents had heard moderate to a great deal about George Floyd's death, and nearly a third of respondents were not satisfied with how Minnesota state and local government officials handled the investigation into his death. Among Black respondents, greater dissatisfaction in the response to his death was associated with lower trustworthiness ratings for pharmaceutical companies, the Trump/Pence administration, and state elected officials. Among Hispanic respondents, greater dissatisfaction in the response to George Floyd's death was associated with lower trustworthiness ratings of the Trump Administration and state elected officials, exclusively. Previous work has found that individual and vicarious experiences with police officers may fuel general mistrust of law enforcement and governmental actors.^84^ While George Floyd's death may represent a more salient contemporary event, many speculate that harmful historical events also shape perceptions of the contemporary trustworthiness of government institutions.^76^

These events are only a few of many recent and historical circumstances and events that may influence public trust in government, public health, and medical institutions. While we found lowered levels of trust in government officials and administrations, we did not find evidence of the erosion of trust in direct sources of health care delivery, information, or regulation: usual sources of care, vaccine clinics, or the FDA. This may be due to a number of factors—the perceived nonpolitical nature of healthcare, the increased interpersonal interactions within healthcare, the duration of relationships within healthcare, or other factors. One meta‐analysis of 39 studies found trust often increases with the duration of relationships and frequency of contact.^85^

### 4.1 Limitations

First, our data are cross‐sectional and were collected using a non‐probabilistic online sample. Therefore, our estimates are not nationally representative and likely include bias due to coverage error associated with the sampling technique and mode of survey administration. We were also unable to determine the extent of survey non‐response bias. Participants in our study, however, represent a diverse group of adults from all regions of the United States. To our knowledge, our study is the largest national sample of Black and Hispanic Americans who were asked about their knowledge of these historical and contemporary events. Second, we did not include community‐based organizations and other trusted community settings where people were able to obtain vaccines. Finally, we also used single‐item measures of trust that, while novel and potentially important, have not been validated or psychometrically tested.

## 5 CONCLUSION

Trust in public and private institutions depends on their trustworthiness. Public health practitioners rely on a wellspring of trust in emergencies when the general population must act quickly (e.g., using masks and taking vaccines) to stop the spread of disease. But this trust must be earned by improving the trustworthiness of institutions. The erosion of trust through untrustworthy actions of one institution or system (e.g., the criminal legal system) may spill over to other distinct institutions and systems (e.g., health care).^47^ Our findings suggest an association between perceptions of trustworthiness in the people and institutions involved in the development and distribution of coronavirus vaccines and events that erode trust in American institutions. Trustworthiness varied between actors in government and health care institutions with differential associations with historical and contemporary atrocities among Black and Hispanic respondents. Respondents generally rated their doctors and health care teams, pharmacies and walk‐in clinics, and the FDA as trustworthy. They rated elected officials and the Biden and Trump administrations as less trustworthy. Future research should assess whether similar contemporary experiences within and beyond health systems have an impact on trust in doctors and health care teams. Improving the trustworthiness of American institutions for Black and Hispanic populations in the U.S. is not only essential to an improved response to future pandemics but also to creating cultures of health that produce equitable outcomes for all populations.

## Supporting information


**Appendix A.** Supporting information.

**Appendix B.** COVID‐19 Pandemic Trust, Racism, and State Violence Survey.
