# Supplementary material for: Public trust is earned: Historical discrimination, carceral violence, and the COVID‐19 pandemic
Source: Health Serv Res. 2023 Jun 6;58(Suppl 2):218–28. doi: 10.1111/1475-6773.14187 (PMC10339167; doi:10.1111/1475-6773.14187)
Supplement: Supplementary file 1 — Appendix A. Supporting information. [file HESR-58-218-s001.docx]

| **Supplemental Appendix A**: Association between trust and knowledge of high profile carceral violence and historical atrocities among **Black Respondents** | | | | | | | | | | | | | | |
| --- | --- | --- | --- | --- | --- | --- | --- | --- | --- | --- | --- | --- | --- | --- |
|  | Pharma | | FDA | | Trump Administration | | Biden Administration | | Usual source of care | | Vaccine | | State Elected Officials | |
|  | b | ci95 | b | ci95 | b | ci95 | b | ci95 | b | ci95 | b | ci95 | b | ci95 |
| Knowledge of George Floyd - Great deal/a lot/moderate (ref= little/none) | 0.06 | [-0.0,0.2] | 0.07 | [-0.0,0.2] | 0.04 | [-0.1,0.1] | 0.05 | [-0.0,0.1] | 0.13** | [0.0,0.2] | 0.05 | [-0.0,0.1] | 0.07 | [-0.0,0.2] |
| Knowledge of Tuskegee - Great deal/a lot/moderate (ref= little/none) | 0.04 | [-0.0,0.1] | 0.01 | [-0.1,0.1] | 0.05 | [-0.0,0.1] | 0.06 | [-0.0,0.1] | 0.09** | [0.0,0.2] | 0.03 | [-0.0,0.1] | 0.07 | [-0.0,0.1] |
| Knowledge of U.S. Immigration and Customs Enforcement - Great deal/a lot/moderate (ref= little/none) | -0.05 | [-0.1,0.0] | 0 | [-0.1,0.1] | -0.05 | [-0.1,0.0] | 0 | [-0.1,0.1] | 0.02 | [-0.0,0.1] | -0.01 | [-0.1,0.0] | -0.04 | [-0.1,0.0] |
| Shared destiny - Strongly agree/agree (ref= little/none) | 0.04 | [-0.0,0.1] | 0.06* | [0.0,0.1] | -0.03 | [-0.1,0.0] | 0.06* | [0.0,0.1] | 0.03 | [-0.0,0.1] | 0.01 | [-0.0,0.1] | 0.02 | [-0.0,0.1] |
| Age 35-49 (ref =18-34) | 0.02 | [-0.0,0.1] | 0 | [-0.1,0.1] | -0.01 | [-0.1,0.1] | 0.06 | [-0.0,0.1] | 0.04 | [-0.0,0.1] | 0.03 | [-0.0,0.1] | -0.01 | [-0.1,0.1] |
| Age 50-64 (ref =18-34) | 0.09* | [0.0,0.2] | 0.07* | [0.0,0.1] | -0.11* | [-0.2,-0.0] | 0.15*** | [0.1,0.2] | 0.11*** | [0.0,0.2] | 0.13*** | [0.1,0.2] | 0.05 | [-0.0,0.1] |
| Age 65+ (ref =18-34) | 0.13** | [0.0,0.2] | 0.14*** | [0.1,0.2] | -0.16** | [-0.3,-0.1] | 0.18*** | [0.1,0.3] | 0.09 | [-0.0,0.2] | 0.13** | [0.1,0.2] | 0.04 | [-0.1,0.2] |
| Female (ref: male) | -0.02 | [-0.1,0.0] | -0.04 | [-0.1,0.0] | -0.05 | [-0.1,0.0] | 0.02 | [-0.0,0.1] | -0.07** | [-0.1, -0.0] | -0.03 | [-0.1,0.0] | -0.05 | [-0.1,0.0] |
| HS income 25,000-49,999 (ref= <24,999) | 0.04 | [-0.0,0.1] | 0.02 | [-0.0,0.1] | -0.07 | [-0.1,0.0] | 0.05 | [-0.0,0.1] | 0.04 | [-0.0,0.1] | -0.01 | [-0.1,0.0] | 0.05 | [-0.0,0.1] |
| HS income $50,000-99,999 (ref= <24,999) | 0.06 | [-0.0,0.2] | 0.09* | [0.0,0.2] | -0.01 | [-0.1,0.1] | 0.11** | [0.0,0.2] | 0.02 | [-0.1,0.1] | -0.01 | [-0.1,0.1] | 0.13* | [0.0,0.2] |
| HS income 100,000-199,000 (ref= <24,999) | 0.04 | [-0.1,0.1] | 0.13*** | [0.1,0.2] | -0.01 | [-0.1,0.1] | 0.07 | [-0.0,0.2] | 0.08 | [-0.0,0.2] | 0.06 | [-0.0,0.1] | 0.03 | [-0.1,0.1] |
| HS income 200,000+ (ref= <24,999) | 0.04 | [-0.1,0.2] | -0.13 | [-0.3,0.1] | -0.1 | [-0.3,0.1] | 0.18** | [0.1,0.3] | 0.16*** | [0.1,0.2] | 0.08 | [-0.0,0.2] | 0.17 | [-0.0,0.4] |
| High School/GED Education (ref= less than High School) | 0.02 | [-0.1,0.1] | 0.03 | [-0.1,0.1] | -0.11 | [-0.2,0.0] | -0.02 | [-0.1,0.1] | 0.03 | [-0.1,0.1] | 0.06 | [-0.0,0.2] | 0.05 | [-0.1,0.2] |
| Some college, no degree (ref= less than High School) | 0.01 | [-0.1,0.1] | 0.05 | [-0.1,0.2] | -0.11 | [-0.2,0.0] | -0.02 | [-0.1,0.1] | 0.05 | [-0.1,0.1] | 0.06 | [-0.1,0.2] | 0.06 | [-0.1,0.2] |
| College degree or more (ref= less than High School) | 0 | [-0.1,0.1] | 0 | [-0.1,0.1] | -0.13 | [-0.3,0.0] | -0.05 | [-0.1,0.0] | 0.01 | [-0.1,0.1] | 0.07 | [-0.0,0.2] | -0.01 | [-0.1,0.1] |
| Medicaid (ref = private) | -0.03 | [-0.1,0.0] | -0.01 | [-0.1,0.1] | 0.02 | [-0.1,0.1] | -0.05 | [-0.1,0.0] | 0.01 | [-0.1,0.1] | -0.04 | [-0.1,0.0] | 0.03 | [-0.1,0.1] |
| Medicare (ref = private) | 0.03 | [-0.1,0.1] | -0.01 | [-0.1,0.1] | 0.08 | [-0.0,0.2] | -0.02 | [-0.1,0.1] | 0.03 | [-0.0,0.1] | -0.01 | [-0.1,0.1] | 0.11* | [0.0,0.2] |
| Other (ref = private) | -0.02 | [-0.1,0.1] | -0.02 | [-0.1,0.1] | -0.01 | [-0.1,0.1] | -0.12** | [-0.2,-0.0] | 0 | [-0.1,0.1] | -0.06 | [-0.1,0.0] | 0.04 | [-0.1,0.1] |
| Uninsured (ref = private) | -0.12 | [-0.2,0.0] | -0.13* | [-0.2,-0.0] | 0 | [-0.1,0.1] | -0.11* | [-0.2,-0.0] | -0.07 | [-0.2,0.0] | -0.12* | [-0.2,-0.0] | -0.19** | [-0.3,-0.1] |
| US born (ref= not US born) | -0.11* | [-0.2,-0.0] | -0.03 | [-0.1,0.1] | -0.07 | [-0.2,0.1] | -0.08 | [-0.2,0.0] | 0.02 | [-0.1,0.1] | 0 | [-0.1,0.1] | 0 | [-0.2,0.2] |
| Good Physical Health (ref= excellent/very good) | 0.07* | [0.0,0.1] | 0.07* | [0.0,0.1] | -0.02 | [-0.1,0.1] | 0.02 | [-0.0,0.1] | 0.08** | [0.0,0.1] | 0.07* | [0.0,0.1] | 0.01 | [-0.1,0.1] |
| Fair/poor Physical Health (ref= excellent/very good) | 0 | [-0.1,0.1] | 0.07 | [-0.0,0.1] | -0.11* | [-0.2,-0.0] | 0.04 | [-0.0,0.1] | 0.08* | [0.0,0.2] | 0.08* | [0.0,0.2] | -0.05 | [-0.1,0.1] |
| Good Mental Health (ref= excellent/very good) | 0 | [-0.1,0.1] | 0.02 | [-0.0,0.1] | -0.10* | [-0.2,-0.0] | 0.04 | [-0.0,0.1] | -0.08* | [-0.1,-0.0] | -0.01 | [-0.1,0.1] | 0 | [-0.1,0.1] |
| Fair/poor Mental Health (ref= excellent/very good) | -0.03 | [-0.1,0.0] | -0.01 | [-0.1,0.1] | -0.08 | [-0.2,0.0] | 0.02 | [-0.1,0.1] | -0.04 | [-0.1,0.0] | 0 | [-0.1,0.1] | -0.08 | [-0.2,0.0] |
| Democrat (ref= republican) | 0.08 | [-0.0,0.2] | 0.07 | [-0.0,0.2] | -0.38*** | [-0.5,-0.3] | 0.31*** | [0.2,0.4] | 0.01 | [-0.1,0.1] | 0.04 | [-0.1,0.1] | -0.04 | [-0.2,0.1] |
| Independent (ref= republican) | -0.05 | [-0.2,0.1] | 0.02 | [-0.1,0.1] | -0.31*** | [-0.5,-0.2] | 0.14* | [0.0,0.3] | -0.03 | [-0.1,0.1] | -0.04 | [-0.1,0.1] | -0.18** | [-0.3,-0.0] |
| Something else (ref= republican) | 0.03 | [-0.1,0.2] | -0.01 | [-0.2,0.1] | -0.34*** | [-0.5,-0.2] | 0.11 | [-0.0,0.3] | 0 | [-0.1,0.1] | -0.03 | [-0.2,0.1] | -0.11 | [-0.3,0.1] |

| **Supplemental Appendix B:** Association between trust and knowledge of high profile carceral violence and historical atrocities among **Hispanic Respondents** | | | | | | | | | | | | | | |
| --- | --- | --- | --- | --- | --- | --- | --- | --- | --- | --- | --- | --- | --- | --- |
|  | Pharma | | FDA | | Trump Administration | | Biden Administration | | Usual source of care | | Vaccine | | State Elected Officials | |
|  | b | ci95 | b | ci95 | b | ci95 | b | ci95 | b | ci95 | b | ci95 | b | ci95 |
| Knowledge of George Floyd - Great deal/a lot/moderate (ref= little/none) | 0 | [0.0,0.0] | 0 | [0.0,0.0] | 0 | [0.0,0.0] | 0 | [0.0,0.0] | 0 | [0.0,0.0] | 0 | [0.0,0.0] | 0 | [0.0,0.0] |
| Knowledge of Tuskegee - Great deal/a lot/moderate (ref= little/none) | -0.07 | [-0.1,0.0] | -0.04 | [-0.1,0.0] | 0.11* | [0.0,0.2] | -0.04 | [-0.1,0.0] | -0.04 | [-0.1,0.0] | -0.03 | [-0.1,0.0] | -0.01 | [-0.1,0.1] |
| Knowledge of U.S. Immigration and Customs Enforcement - Great deal/a lot/moderate (ref= little/none) | -0.09** | [-0.2,-0.0] | -0.07* | [-0.1,-0.0] | -0.09** | [-0.2,-0.0] | -0.08* | [-0.1,-0.0] | -0.07* | [-0.1,-0.0] | -0.03 | [-0.1,0.0] | -0.11** | [-0.2,-0.0] |
| Shared destiny - Strongly agree/agree (ref= little/none) | 0 | [0.0,0.0] | 0 | [0.0,0.0] | 0 | [0.0,0.0] | 0 | [0.0,0.0] | 0 | [0.0,0.0] | 0 | [0.0,0.0] | 0 | [0.0,0.0] |
| Age 35-49 (ref =18-34) | 0.04 | [-0.0,0.1] | 0.06* | [0.0,0.1] | 0 | [-0.1,0.1] | 0.06* | [0.0,0.1] | 0.03 | [-0.0,0.1] | 0.01 | [-0.0,0.1] | 0.04 | [-0.0,0.1] |
| Age 50-64 (ref =18-34) | 0 | [0.0,0.0] | 0 | [0.0,0.0] | 0 | [0.0,0.0] | 0 | [0.0,0.0] | 0 | [0.0,0.0] | 0 | [0.0,0.0] | 0 | [0.0,0.0] |
| Age 65+ (ref =18-34) | 0.03 | [-0.0,0.1] | 0 | [-0.1,0.1] | -0.02 | [-0.1,0.1] | 0.07 | [-0.0,0.1] | 0.02 | [-0.0,0.1] | 0.03 | [-0.0,0.1] | -0.02 | [-0.1,0.1] |
| Female (ref: male) | 0.08* | [0.0,0.2] | 0.08* | [0.0,0.2] | -0.11* | [-0.2,-0.0] | 0.16*** | [0.1,0.2] | 0.09** | [0.0,0.2] | 0.11*** | [0.0,0.2] | 0.04 | [-0.0,0.1] |
| HS income 25,000-49,999 (ref= <24,999) | 0.12** | [0.0,0.2] | 0.13*** | [0.1,0.2] | -0.18*** | [-0.3,-0.1] | 0.16*** | [0.1,0.2] | 0.05 | [-0.1,0.1] | 0.11** | [0.0,0.2] | 0.02 | [-0.1,0.1] |
| HS income $50,000-99,999 (ref= <24,999) | 0 | [0.0,0.0] | 0 | [0.0,0.0] | 0 | [0.0,0.0] | 0 | [0.0,0.0] | 0 | [0.0,0.0] | 0 | [0.0,0.0] | 0 | [0.0,0.0] |
| HS income 100,000-199,000 (ref= <24,999) | -0.01 | [-0.1,0.1] | -0.03 | [-0.1,0.0] | -0.04 | [-0.1,0.0] | 0.04 | [-0.0,0.1] | -0.06* | [-0.1,-0.0] | -0.03 | [-0.1,0.0] | -0.03 | [-0.1,0.0] |
| HS income 200,000+ (ref= <24,999) | 0 | [0.0,0.0] | 0 | [0.0,0.0] | 0 | [0.0,0.0] | 0 | [0.0,0.0] | 0 | [0.0,0.0] | 0 | [0.0,0.0] | 0 | [0.0,0.0] |
| High School/GED Education (ref= less than High School) | 0.02 | [-0.0,0.1] | 0.01 | [-0.1,0.1] | -0.07 | [-0.1,0.0] | 0.03 | [-0.0,0.1] | 0.05 | [-0.0,0.1] | -0.02 | [-0.1,0.0] | 0.04 | [-0.0,0.1] |
| Some college, no degree (ref= less than High School) | 0.03 | [-0.1,0.1] | 0.07 | [-0.0,0.2] | -0.06 | [-0.2,0.0] | 0.09* | [0.0,0.2] | 0.03 | [-0.1,0.1] | -0.01 | [-0.1,0.1] | 0.1 | [-0.0,0.2] |
| College degree or more (ref= less than High School) | 0.02 | [-0.1,0.1] | 0.11** | [0.0,0.2] | -0.01 | [-0.1,0.1] | 0.06 | [-0.0,0.1] | 0.09* | [0.0,0.2] | 0.05 | [-0.0,0.1] | 0.01 | [-0.1,0.1] |
| Medicaid (ref = private) | 0.09 | [-0.1,0.2] | -0.12 | [-0.3,0.1] | -0.04 | [-0.3,0.2] | 0 | [0.0,0.0] | 0 | [0.0,0.0] | 0.09 | [-0.0,0.2] | 0.17 | [-0.0,0.4] |
| Medicare (ref = private) | 0 | [0.0,0.0] | 0 | [0.0,0.0] | 0 | [0.0,0.0] | 0 | [0.0,0.0] | 0 | [0.0,0.0] | 0 | [0.0,0.0] | 0 | [0.0,0.0] |
| Other (ref = private) | -0.01 | [-0.1,0.1] | 0.01 | [-0.1,0.1] | -0.14 | [-0.3,0.0] | -0.04 | [-0.1,0.1] | 0.01 | [-0.1,0.1] | 0.04 | [-0.1,0.1] | 0.03 | [-0.1,0.2] |
| Uninsured (ref = private) | -0.01 | [-0.1,0.1] | 0.03 | [-0.1,0.1] | -0.12 | [-0.3,0.0] | -0.02 | [-0.1,0.1] | 0.04 | [-0.1,0.1] | 0.04 | [-0.1,0.1] | 0.06 | [-0.1,0.2] |
| US born (ref= not US born) | -0.02 | [-0.1,0.1] | -0.02 | [-0.1,0.1] | -0.13 | [-0.3,0.0] | -0.05 | [-0.1,0.0] | 0.02 | [-0.1,0.1] | 0.06 | [-0.1,0.2] | 0 | [-0.1,0.1] |
| Good Physical Health (ref= excellent/very good) | 0 | [0.0,0.0] | 0 | [0.0,0.0] | 0 | [0.0,0.0] | 0 | [0.0,0.0] | 0 | [0.0,0.0] | 0 | [0.0,0.0] | 0 | [0.0,0.0] |
| Fair/poor Physical Health (ref= excellent/very good) | -0.02 | [-0.1,0.1] | -0.02 | [-0.1,0.1] | 0.01 | [-0.1,0.1] | -0.05 | [-0.1,0.0] | 0 | [-0.1,0.1] | -0.03 | [-0.1,0.0] | 0.02 | [-0.1,0.1] |
| Good Mental Health (ref= excellent/very good) | 0.03 | [-0.1,0.1] | -0.01 | [-0.1,0.1] | 0.10* | [0.0,0.2] | 0 | [-0.1,0.1] | 0.05 | [-0.0,0.1] | 0.01 | [-0.1,0.1] | 0.11* | [0.0,0.2] |
| Fair/poor Mental Health (ref= excellent/very good) | -0.01 | [-0.1,0.1] | -0.02 | [-0.1,0.1] | 0 | [-0.1,0.1] | -0.10* | [-0.2,-0.0] | -0.01 | [-0.1,0.1] | -0.03 | [-0.1,0.0] | 0.04 | [-0.1,0.1] |
| Democrat (ref= republican) | -0.12 | [-0.2,0.0] | -0.15* | [-0.3,-0.0] | -0.01 | [-0.1,0.1] | -0.11 | [-0.2,0.0] | -0.07 | [-0.2,0.0] | -0.11 | [-0.2,0.0] | -0.20** | [-0.3,-0.1] |
| Independent (ref= republican) | 0 | [0.0,0.0] | 0 | [0.0,0.0] | 0 | [0.0,0.0] | 0 | [0.0,0.0] | 0 | [0.0,0.0] | 0 | [0.0,0.0] | 0 | [0.0,0.0] |
| Something else (ref= republican) | -0.12* | [-0.2,-0.0] | -0.04 | [-0.2,0.1] | -0.09 | [-0.3,0.1] | -0.08 | [-0.2,0.0] | 0.03 | [-0.1,0.2] | 0 | [-0.1,0.1] | 0.01 | [-0.2,0.2] |

| **Supplemental Appendix C:** Association between trust and satisfaction with George Floyd Investigation among **Black Respondents** | | | | | | | | | | | | | | |
| --- | --- | --- | --- | --- | --- | --- | --- | --- | --- | --- | --- | --- | --- | --- |
|  | Pharma | | FDA | | Trump Administration | | Biden Administration | | Usual source of care | | Vaccine | | State Elected Officials | |
|  | b | ci95 | b | ci95 | b | ci95 | b | ci95 | b | ci95 | b | ci95 | b | ci95 |
| Neutral satisfaction with George Floyd Investigation (reference= satisfied) | -0.07 | [-0.1,0.0] | -0.04 | [-0.1,0.0] | 0.11* | [0.0,0.2] | -0.04 | [-0.1,0.0] | -0.04 | [-0.1,0.0] | -0.03 | [-0.1,0.0] | -0.01 | [-0.1,0.1] |
| Dissatisfaction with George Floyd Investigation (reference= satisfied) | -0.09** | [-0.2,-0.0] | -0.07* | [-0.1,-0.0] | -0.09** | [-0.2,-0.0] | -0.08* | [-0.1,-0.0] | -0.07* | [-0.1,-0.0] | -0.03 | [-0.1,0.0] | -0.11** | [-0.2,-0.0] |
| Shared destiny - Strongly agree/agree (ref= little/none) | 0.04 | [-0.0,0.1] | 0.06* | [0.0,0.1] | 0 | [-0.1,0.1] | 0.06* | [0.0,0.1] | 0.03 | [-0.0,0.1] | 0.01 | [-0.0,0.1] | 0.04 | [-0.0,0.1] |
| Age 35-49 (ref =18-34) | 0.03 | [-0.0,0.1] | 0 | [-0.1,0.1] | -0.02 | [-0.1,0.1] | 0.07 | [-0.0,0.1] | 0.02 | [-0.0,0.1] | 0.03 | [-0.0,0.1] | -0.02 | [-0.1,0.1] |
| Age 50-64 (ref =18-34) | 0.08* | [0.0,0.2] | 0.08* | [0.0,0.2] | -0.11* | [-0.2,-0.0] | 0.16*** | [0.1,0.2] | 0.09** | [0.0,0.2] | 0.11*** | [0.0,0.2] | 0.04 | [-0.0,0.1] |
| Age 65+ (ref =18-34) | 0.12** | [0.0,0.2] | 0.13*** | [0.1,0.2] | -0.18*** | [-0.3,-0.1] | 0.16*** | [0.1,0.2] | 0.05 | [-0.1,0.1] | 0.11** | [0.0,0.2] | 0.02 | [-0.1,0.1] |
| Female (ref: male) | -0.01 | [-0.1,0.1] | -0.03 | [-0.1,0.0] | -0.04 | [-0.1,0.0] | 0.04 | [-0.0,0.1] | -0.06* | [-0.1,-0.0] | -0.03 | [-0.1,0.0] | -0.03 | [-0.1,0.0] |
| HS income 25,000-49,999 (ref= <24,999) | 0.02 | [-0.0,0.1] | 0.01 | [-0.1,0.1] | -0.07 | [-0.1,0.0] | 0.03 | [-0.0,0.1] | 0.05 | [-0.0,0.1] | -0.02 | [-0.1,0.0] | 0.04 | [-0.0,0.1] |
| HS income $50,000-99,999 (ref= <24,999) | 0.03 | [-0.1,0.1] | 0.07 | [-0.0,0.2] | -0.06 | [-0.2,0.0] | 0.09* | [0.0,0.2] | 0.03 | [-0.1,0.1] | -0.01 | [-0.1,0.1] | 0.1 | [-0.0,0.2] |
| HS income 100,000-199,000 (ref= <24,999) | 0.02 | [-0.1,0.1] | 0.11** | [0.0,0.2] | -0.01 | [-0.1,0.1] | 0.06 | [-0.0,0.1] | 0.09* | [0.0,0.2] | 0.05 | [-0.0,0.1] | 0.01 | [-0.1,0.1] |
| HS income 200,000+ (ref= <24,999) | 0.09 | [-0.1,0.2] | -0.12 | [-0.3,0.1] | -0.04 | [-0.3,0.2] | 0 | [0.0,0.0] | 0 | [0.0,0.0] | 0.09 | [-0.0,0.2] | 0.17 | [-0.0,0.4] |
| High School/GED Education (ref= less than High School) | -0.01 | [-0.1,0.1] | 0.01 | [-0.1,0.1] | -0.14 | [-0.3,0.0] | -0.04 | [-0.1,0.1] | 0.01 | [-0.1,0.1] | 0.04 | [-0.1,0.1] | 0.03 | [-0.1,0.2] |
| Some college, no degree (ref= less than High School) | -0.01 | [-0.1,0.1] | 0.03 | [-0.1,0.1] | -0.12 | [-0.3,0.0] | -0.02 | [-0.1,0.1] | 0.04 | [-0.1,0.1] | 0.04 | [-0.1,0.1] | 0.06 | [-0.1,0.2] |
| College degree or more (ref= less than High School) | -0.02 | [-0.1,0.1] | -0.02 | [-0.1,0.1] | -0.13 | [-0.3,0.0] | -0.05 | [-0.1,0.0] | 0.02 | [-0.1,0.1] | 0.06 | [-0.1,0.2] | 0 | [-0.1,0.1] |
| Medicaid (ref = private) | -0.02 | [-0.1,0.1] | -0.02 | [-0.1,0.1] | 0.01 | [-0.1,0.1] | -0.05 | [-0.1,0.0] | 0 | [-0.1,0.1] | -0.03 | [-0.1,0.0] | 0.02 | [-0.1,0.1] |
| Medicare (ref = private) | 0.03 | [-0.1,0.1] | -0.01 | [-0.1,0.1] | 0.10* | [0.0,0.2] | 0 | [-0.1,0.1] | 0.05 | [-0.0,0.1] | 0.01 | [-0.1,0.1] | 0.11* | [0.0,0.2] |
| Other (ref = private) | -0.01 | [-0.1,0.1] | -0.02 | [-0.1,0.1] | 0 | [-0.1,0.1] | -0.10* | [-0.2,-0.0] | -0.01 | [-0.1,0.1] | -0.03 | [-0.1,0.0] | 0.04 | [-0.1,0.1] |
| Uninsured (ref = private) | -0.12 | [-0.2,0.0] | -0.15* | [-0.3,-0.0] | -0.01 | [-0.1,0.1] | -0.11 | [-0.2,0.0] | -0.07 | [-0.2,0.0] | -0.11 | [-0.2,0.0] | -0.20** | [-0.3,-0.1] |
| US born (ref= not US born) | -0.12* | [-0.2,-0.0] | -0.04 | [-0.2,0.1] | -0.09 | [-0.3,0.1] | -0.08 | [-0.2,0.0] | 0.03 | [-0.1,0.2] | 0 | [-0.1,0.1] | 0.01 | [-0.2,0.2] |
| Good Physical Health (ref= excellent/very good) | 0.06 | [-0.0,0.1] | 0.07* | [0.0,0.1] | -0.02 | [-0.1,0.1] | 0.01 | [-0.1,0.1] | 0.08* | [0.0,0.1] | 0.06 | [-0.0,0.1] | 0.01 | [-0.1,0.1] |
| Fair/poor Physical Health (ref= excellent/very good) | 0 | [-0.1,0.1] | 0.08 | [-0.0,0.2] | -0.10* | [-0.2,-0.0] | 0.04 | [-0.0,0.1] | 0.09* | [0.0,0.2] | 0.08* | [0.0,0.2] | -0.04 | [-0.1,0.1] |
| Good Mental Health (ref= excellent/very good) | 0.01 | [-0.1,0.1] | 0.02 | [-0.0,0.1] | -0.11** | [-0.2,-0.0] | 0.04 | [-0.0,0.1] | -0.10** | [-0.2,-0.0] | -0.01 | [-0.1,0.1] | 0 | [-0.1,0.1] |
| Fair/poor Mental Health (ref= excellent/very good) | -0.01 | [-0.1,0.1] | 0.01 | [-0.1,0.1] | -0.09 | [-0.2,0.0] | 0.02 | [-0.1,0.1] | -0.07 | [-0.1,0.0] | 0 | [-0.1,0.1] | -0.08 | [-0.2,0.0] |
| Democrat (ref= republican) | 0.04 | [-0.1,0.1] | 0.03 | [-0.1,0.1] | -0.44*** | [-0.6,-0.3] | 0.31*** | [0.2,0.4] | -0.03 | [-0.1,0.1] | 0 | [-0.1,0.1] | -0.1 | [-0.2,0.0] |
| Independent (ref= republican) | -0.09 | [-0.2,0.0] | -0.02 | [-0.1,0.1] | -0.39*** | [-0.5,-0.3] | 0.14 | [-0.0,0.3] | -0.04 | [-0.1,0.1] | -0.07 | [-0.2,0.0] | -0.24*** | [-0.4,-0.1] |
| Something else (ref= republican) | 0 | [-0.1,0.1] | -0.06 | [-0.2,0.1] | -0.40*** | [-0.6,-0.2] | 0.14 | [-0.0,0.3] | -0.04 | [-0.2,0.1] | -0.06 | [-0.2,0.1] | -0.16* | [-0.3,-0.0] |

| **Supplemental Appendix D**: Association between trust and satisfaction with George Floyd Investigation among **Hispanic Respondents** | | | | | | | | | | | | | | |
| --- | --- | --- | --- | --- | --- | --- | --- | --- | --- | --- | --- | --- | --- | --- |
|  | Pharma | | FDA | | Trump Administration | | Biden Administration | | Usual source of care | | Vaccine | | State Elected Officials | |
|  | b | ci95 | b | ci95 | b | ci95 | b | ci95 | b | ci95 | b | ci95 | b | ci95 |
| Neutral satisfaction with George Floyd Investigation (reference= satisfied) | -0.03 | [-0.1,0.0] | -0.03 | [-0.1,0.0] | 0.05 | [-0.0,0.1] | -0.08* | [-0.1,-0.0] | 0.03 | [-0.0,0.1] | -0.05 | [-0.1,0.0] | 0 | [-0.1,0.1] |
| Dissatisfaction with George Floyd Investigation (reference= satisfied) | -0.05 | [-0.1,0.0] | -0.03 | [-0.1,0.0] | -0.15*** | [-0.2, -0.1] | -0.02 | [-0.1,0.1] | 0.03 | [-0.0,0.1] | 0.01 | [-0.0,0.1] | -0.11** | [-0.2, -0.0] |
| Shared destiny - Strongly agree/agree (ref= little/none) | 0 | [-0.1,0.1] | 0.06* | [0.0,0.1] | -0.02 | [-0.1,0.0] | 0.09** | [0.0,0.2] | 0.05 | [-0.0,0.1] | 0.02 | [-0.0,0.1] | 0.12*** | [0.0,0.2] |
| Age 35-49 (ref =18-34) | 0 | [-0.1,0.1] | 0 | [-0.1,0.1] | 0.01 | [-0.1,0.1] | 0.02 | [-0.0,0.1] | -0.02 | [-0.1,0.0] | 0.04 | [-0.0,0.1] | 0.02 | [-0.1,0.1] |
| Age 50-64 (ref =18-34) | 0 | [-0.1,0.1] | 0.02 | [-0.1,0.1] | -0.13* | [-0.2,-0.0] | -0.01 | [-0.1,0.1] | 0 | [-0.1,0.1] | 0.01 | [-0.1,0.1] | -0.11* | [-0.2,-0.0] |
| Age 65+ (ref =18-34) | 0.07 | [-0.0,0.2] | 0.13*** | [0.1,0.2] | -0.19** | [-0.3,-0.0] | 0.07 | [-0.0,0.2] | 0.06 | [-0.0,0.2] | 0.08 | [-0.0,0.2] | -0.11 | [-0.3,0.1] |
| Female (ref: male) | 0.03 | [-0.0,0.1] | 0 | [-0.1,0.0] | -0.05 | [-0.1,0.0] | 0.01 | [-0.0,0.1] | 0.04 | [-0.0,0.1] | 0.03 | [-0.0,0.1] | 0.04 | [-0.0,0.1] |
| HS income 25,000-49,999 (ref= <24,999) | 0.03 | [-0.0,0.1] | -0.03 | [-0.1,0.0] | 0.01 | [-0.1,0.1] | -0.05 | [-0.1,0.0] | 0.03 | [-0.0,0.1] | -0.07* | [-0.1,-0.0] | -0.01 | [-0.1,0.1] |
| HS income $50,000-99,999 (ref= <24,999) | 0.01 | [-0.1,0.1] | -0.04 | [-0.1,0.0] | 0.13* | [0.0,0.2] | -0.04 | [-0.1,0.1] | 0.08 | [-0.0,0.2] | -0.07 | [-0.2,0.0] | -0.08 | [-0.2,0.0] |
| HS income 100,000-199,000 (ref= <24,999) | 0.11** | [0.0,0.2] | 0.06 | [-0.0,0.1] | 0.11 | [-0.0,0.2] | 0.09* | [0.0,0.2] | 0.13** | [0.0,0.2] | 0.01 | [-0.1,0.1] | 0.08 | [-0.0,0.2] |
| HS income 200,000+ (ref= <24,999) | 0.01 | [-0.2,0.2] | -0.17 | [-0.5,0.1] | 0.16 | [-0.1,0.5] | 0.06 | [-0.2,0.3] | 0.03 | [-0.2,0.3] | 0.03 | [-0.2,0.2] | 0.07 | [-0.2,0.4] |
| High School/GED Education (ref= less than High School) | -0.05 | [-0.2,0.0] | 0.13 | [-0.0,0.3] | 0.07 | [-0.1,0.2] | -0.04 | [-0.2,0.1] | 0.01 | [-0.1,0.1] | -0.02 | [-0.1,0.1] | -0.1 | [-0.2,0.0] |
| Some college, no degree (ref= less than High School) | -0.05 | [-0.2,0.0] | 0.17* | [0.0,0.3] | 0.04 | [-0.1,0.2] | -0.04 | [-0.2,0.1] | 0 | [-0.1,0.1] | 0.03 | [-0.1,0.1] | -0.02 | [-0.2,0.1] |
| College degree or more (ref= less than High School) | -0.03 | [-0.1,0.1] | 0.14 | [-0.0,0.3] | 0.04 | [-0.1,0.2] | -0.03 | [-0.1,0.1] | -0.02 | [-0.1,0.1] | 0.02 | [-0.1,0.1] | -0.01 | [-0.2,0.1] |
| Medicaid (ref = private) | 0.01 | [-0.1,0.1] | 0.04 | [-0.0,0.1] | 0.12* | [0.0,0.2] | 0.01 | [-0.1,0.1] | -0.03 | [-0.1,0.0] | -0.08 | [-0.2,0.0] | 0.09 | [-0.0,0.2] |
| Medicare (ref = private) | -0.05 | [-0.1,0.0] | -0.05 | [-0.1,0.0] | 0.09 | [-0.0,0.2] | -0.03 | [-0.1,0.1] | -0.06 | [-0.1,0.0] | -0.05 | [-0.1,0.0] | 0.12* | [0.0,0.2] |
| Other (ref = private) | -0.08 | [-0.2,0.0] | 0.01 | [-0.1,0.1] | 0.1 | [-0.0,0.2] | -0.04 | [-0.1,0.1] | -0.08 | [-0.2,0.0] | -0.06 | [-0.1,0.0] | 0.06 | [-0.0,0.2] |
| Uninsured (ref = private) | -0.05 | [-0.1,0.0] | 0.05 | [-0.0,0.1] | 0.08 | [-0.0,0.2] | 0.01 | [-0.1,0.1] | -0.13** | [-0.2, -0.0] | -0.02 | [-0.1,0.1] | 0.07 | [-0.0,0.2] |
| US born (ref= not US born) | -0.03 | [-0.1,0.0] | -0.02 | [-0.1,0.0] | -0.06 | [-0.1,0.0] | -0.05 | [-0.1,0.0] | -0.04 | [-0.1,0.0] | -0.01 | [-0.1,0.1] | -0.09* | [-0.2, -0.0] |
| Good Physical Health (ref= excellent/very good) | 0 | [-0.1,0.1] | -0.02 | [-0.1,0.1] | -0.07 | [-0.2,0.0] | -0.01 | [-0.1,0.1] | 0.03 | [-0.0,0.1] | 0.08* | [0.0,0.2] | -0.01 | [-0.1,0.1] |
| Fair/poor Physical Health (ref= excellent/very good) | -0.03 | [-0.1,0.1] | -0.03 | [-0.1,0.1] | -0.04 | [-0.1,0.1] | -0.04 | [-0.1,0.1] | 0.06 | [-0.0,0.1] | 0.05 | [-0.0,0.1] | -0.03 | [-0.1,0.1] |
| Good Mental Health (ref= excellent/very good) | -0.01 | [-0.1,0.1] | 0.03 | [-0.0,0.1] | 0 | [-0.1,0.1] | 0.03 | [-0.0,0.1] | -0.05 | [-0.1,0.0] | -0.07 | [-0.1,0.0] | -0.01 | [-0.1,0.1] |
| Fair/poor Mental Health (ref= excellent/very good) | 0.03 | [-0.0,0.1] | 0.04 | [-0.0,0.1] | -0.14** | [-0.2,-0.0] | 0.02 | [-0.1,0.1] | -0.04 | [-0.1,0.0] | -0.05 | [-0.1,0.0] | -0.05 | [-0.1,0.1] |
| Democrat (ref= republican) | 0.06 | [-0.0,0.1] | 0.10* | [0.0,0.2] | -0.39*** | [-0.5,-0.3] | 0.34*** | [0.2,0.4] | 0.02 | [-0.1,0.1] | 0.06 | [-0.0,0.1] | -0.03 | [-0.1,0.1] |
| Independent (ref= republican) | 0.04 | [-0.1,0.1] | 0.05 | [-0.0,0.1] | -0.20*** | [-0.3,-0.1] | 0.13* | [0.0,0.2] | 0.03 | [-0.1,0.1] | -0.01 | [-0.1,0.1] | -0.12* | [-0.2,-0.0] |
| Something else (ref= republican) | -0.02 | [-0.1,0.1] | 0.03 | [-0.1,0.1] | -0.30*** | [-0.4,-0.2] | 0.17* | [0.0,0.3] | -0.03 | [-0.1,0.1] | -0.07 | [-0.2,0.1] | -0.16* | [-0.3,-0.0] |
