# Supplementary material for: Public trust is earned: Historical discrimination, carceral violence, and the COVID‐19 pandemic
Source: Health Serv Res. 2023 Jun 6;58(Suppl 2):218–28. doi: 10.1111/1475-6773.14187 (PMC10339167; doi:10.1111/1475-6773.14187)
Supplement: Supplementary file 2 — Appendix B. COVID‐19 Pandemic Trust, Racism, and State Violence Survey. [file HESR-58-218-s002.docx]

COVID-19 Pandemic Trust, Racism, and State Violence Survey

Start of Block: Consent Page

Q1 **CONSENT FORM
 This form has information to help you decide whether you want to participate in a study.  If you are interested in participating in the study, please read this form carefully. You will need to consider what the study involves, including what will be required of you and the risks of participation. You may want to discuss the information in the form with a family member or friend. The form tells you the names of people you can contact if you have questions.**
 **Research is not treatment:** The purpose of this research is to assess your intent or willingness to receive the COVID-19 vaccine (and whether you have been vaccinated), trust in medical and governmental institutions involved in the COVID-19 response, experiences with discrimination, and your perceptions of racial violence and social divisions during the pandemic.
 **Whether you participate in research is up to you:** Your participation is completely voluntary.
 **If you participate, it will involve the following:** Participation in this study involves completing an online questionnaire. You will also be asked about your demographic background (e.g., age, sex, and sexual orientation) and questions about your medical history.  You are being asked to participate because you are over 18 years of age and you are currently living in the US.   Your participation is appreciated and important. The information you provide may help us better understand how to improve messaging about COVID-19 vaccines.  If you decide to participate you will be asked to complete an online survey that will take approximately 30 minutes to complete.
 **Participation comes with some risks that you should consider:** The risks to participation in this study are minimal. We are asking questions about issues (e.g., racism and violence) that may cause some discomfort . The information collected through this survey will be completely confidential. No identifiers will be linked to your responses.  Some of the questions may make you uncomfortable or upset, but we have made every attempt to ask questions pertinent to our research questions. There is the potential for loss of confidentiality by participating in the study. Appropriate efforts will be made to protect the confidentiality of your identifiable information as described earlier in this form. There may be other risks that are currently unknown.

 **What is the research study and why is it being done?** The study is being carried out at Tulane University in New Orleans Louisiana.  About 4,000 individuals will participate in the study nationally.  The Tulane University Social/Behavioral Institutional Review Board is an ethics board responsible for overseeing the study at Tulane.
 **Who is paying for the study?** The Robert Wood Johnson Foundation is the study sponsor. 
 **If you take part in this study, how will we protect your privacy?** In this study, your responses to the online questionnaire will be collected.  You will have a study ID number. All of the data we collect from you will be coded with only your ID number and stored in a secure location and on a password-protected secure computer database. The master list linking your study data will be kept in a separate, secure location. We will not identify any individuals who take part in this study in any summaries, reports, or articles that come out of this study.    **What are the possible benefits of being in the study?** You may not receive any direct benefit from participating in the study. We hope that what we learn in this study will give us valuable information about how individuals, families, and communities perceive the governmental response to COVID-19, vaccine development, and the social environment during the pandemic.
 **What will you have to pay for if you take part in the study?** There are no costs to you to participate in the study.
 **Will you be paid to take part in the study?** You will be compensated for completing the questionnaire. Tulane, the study site, or the study site investigator may use information from the study to develop a new product to be sold.  These parties or others may benefit if this happens.  There are no plans to pay you if the information is used for this purpose.
 **What are your other options besides study participation?** You have the option to not participate in the study.  If at any time you wish to leave the study, you may simply close your browser.
 **If you start participating in the study, can you stop later?**  If you participate in the study and want to stop, you can do so at any time.
 **What is done with the results from the study?**  The results of the study may be presented at meetings or in publications.  You will not be identified in any summary, presentation, or publication of the study results without your specific permission. There are no plans to provide either you with information about the results of the study, the results of your individual participation in the study or the results of any research that is not part of the study that may be done as provided in this form, even if those results may be relevant to your health. 
 **What if you have questions or concerns about the study?** If you have any questions or concerns about the study, whether before or after signing this form, you can call any of the people or offices listed below.  You can call about any matter having to do with the study, including complaints or questions about your rights as a study participant.  Study site investigator (researcher):  Andrew Anderson, Ph.D. Phone:  504-988-1939
 If you want to speak with someone who is not directly involved in the study, contact the Tulane University Human Research Protection Office. Phone:  504-988-2665 Email:  irbmain@tulane.edu

- Accept Consent (1)
- Decline Consent (2)

End of Block: Consent Page

Start of Block: Screener Block

Q59 In what state did you primarily reside in 2020?

▼ Alabama (1) ... Wyoming (56)

| 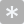 |
| --- |

Q142 What is the zip code of your primary residence in 2020?

________________________________________________________________

| 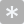 |
| --- |

Q2 What is your age?

________________________________________________________________

Q3 What sex were you assigned at birth?

- Male (1)
- Female (2)
- Other (3)
- Prefer not to say (4)

Q4 To which gender do you most identify?

- Male (1)
- Female (2)
- Transgender Woman (3)
- Transgender Man (4)
- Gender Non-conforming (5)
- Prefer not to say (6)
- Not Listed (7) ________________________________________________

Q137 In the past year, who have you had sex with?

- Men only (1)
- Women only (2)
- Both men and women (3)
- I have not had sex (4)

Q140 In your lifetime, who have you had sex with?

- Men only (1)
- Women only (2)
- Both men and women (3)
- I have not had sex (4)

Q138 Which of the following best describes your sexual orientation?

- Heterosexual or straight (1)
- Gay or lesbian (2)
- Bisexual (3)

Q5 Are you Hispanic/Latinx

- Yes (1)
- No (2)

Q6 Please select your race (select all that apply)

- American Indian/Native American/Alaska Native (1)
- Asian (2)
- Black/African American (3)
- Native Hawaiian or Pacific Islander (4)
- white (5)

Display This Question:

If Please select your race (select all that apply) = American Indian/Native American/Alaska Native

Q7 Are you affiliated with a tribe(s)?

- Yes (enter all tribal affiliations) (1) ________________________________________________
- No (2)

Q39 Please select the Race and/or Ethnicity that you most closely identify with?

- American Indian/Native American/Alaska Native (1)
- Asian (2)
- Black/African American (3)
- Hispanic/Latinx (4)
- Biracial/Multiracial (5)
- Native Hawaiian or Pacific Islander (6)
- White (7)

End of Block: Screener Block

Start of Block: COVID-19 Vaccine Hesitancy Section - Vaccine Receipt

Q9 **We would like to start by asking you questions about your access to a COVID-19 vaccine, your willingness to take it , and your beliefs about the vaccine.**

Q11 Have you already received the COVID-19 vaccine?

- Yes (1)
- No (2)
- No, but I have an appointment to receive it. (3)

Skip To: Q16 If Have you already received the COVID-19 vaccine? != Yes

Skip To: Q13 If Have you already received the COVID-19 vaccine? = Yes

Q13 How many doses have you received?

- One (1)
- Two (2)

Display This Question:

If Have you already received the COVID-19 vaccine? != No

Q135 Are you required to get vaccinated as a condition of maintaining your employment?

- Yes (1)
- No (2)

Display This Question:

If Have you already received the COVID-19 vaccine? = Yes

Q14 When did you receive your first COVID-19 vaccine (i.e. when did you receive your first dose)?

▼ Before December 2020 (1) ... November 2021 (13)

Display This Question:

If Have you already received the COVID-19 vaccine? = Yes

Q15 Which vaccine did you receive?

- Pfizer (1)
- Moderna (2)
- Johnson & Johnson (3)
- I don't know (4)
- Another vaccine (5)

Display This Question:

If Have you already received the COVID-19 vaccine? = No

Q16 How likely are you to get the COVID-19 vaccine?

- Definitely would get it (1)
- Probably would get it (2)
- Probably would not get it (3)
- Definitely would not get it (4)

Q77 Please evaluate the following statement

|  | Strongly Disagree (1) | Disagree (2) | Agree (3) | Strongly Agree (4) |
| --- | --- | --- | --- | --- |
| The benefits of COVID-19 vaccine outweigh the potential risks (1) |  |  |  |  |

Display This Question:

If How likely are you to get the COVID-19 vaccine? = Definitely would not get it

Q18 Briefly, in your own words, what's the main reason why you do not intend to get the COVID-19 vaccine?

________________________________________________________________

Display This Question:

If How likely are you to get the COVID-19 vaccine? = Probably would not get it

Q106 Briefly, in your own words, what's the main reason why you may not get the COVID-19 vaccine?

________________________________________________________________

End of Block: COVID-19 Vaccine Hesitancy Section - Vaccine Receipt

Start of Block: COVID-19 Vaccine Hesitancy - Trust

Q19 Overall, how much do you trust that the coronavirus vaccines are safe?

- Complete (1)
- Mostly (2)
- Somewhat (3)
- Not much (4)
- None at all (5)

Q79 How much do you trust that the COVID-19 vaccines are effective (meaning that they will provide substantial protection against contracting the virus)?

- Complete (1)
- Mostly (2)
- Somewhat (3)
- Not much (4)
- None at all (5)

Q20 Regarding the currently available vaccines for COVID-19, please rate your agreement with the following statements (1= agree completely, 5= completely disagree).

|  | Agree Completely | Completely Disagree |
| --- | --- | --- |

|  | 1 | 2 | 3 | 3 | 4 | 5 |
| --- | --- | --- | --- | --- | --- | --- |

| Early versions of the vaccine are less effective than later versions () | 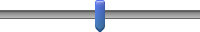 |
| --- | --- |
| There is a possibility of the vaccine itself making people sick () | 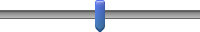 |
| People in your community are getting less safe versions of the vaccine () | 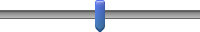 |
| If I get vaccinated for the coronavirus, I think that I will experience side effects () | 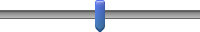 |

End of Block: COVID-19 Vaccine Hesitancy - Trust

Start of Block: Healthcare Mis/Trust Section

Q107 **People have different beliefs about medical care. Thinking about your past experiences or stories you know, we'd like you to share how you feel about doctors, hospitals, nursing homes, and other medical professionals and institutions.**

Q80 How often, if at all, do you...

|  | Every Year (1) | Every Few Years (2) | Less Often (3) | Never (4) |
| --- | --- | --- | --- | --- |
| Get vaccines (e.g., for the seasonal flu)? (1) |  |  |  |  |
| Get other medical treatment recommended by health care providers? (2) |  |  |  |  |

Q81 How often, if at all, do you use natural home remedies to try to prevent or cure illnesses?

- Very Often (1)
- Often (2)
- Sometimes (3)
- Occasionally (4)
- Never (5)

Q83 How much do you agree or disagree with the following statements?

|  | Strongly Disagree (1) | Disagree (2) | Agree (3) | Strongly Agree (4) |
| --- | --- | --- | --- | --- |
| You’d better be cautious when dealing with health care organizations. (1) |  |  |  |  |
| Patients have sometimes been deceived or misled by health care organizations. (2) |  |  |  |  |
| When health care organizations make mistakes they usually cover them up. (3) |  |  |  |  |
| Health care organizations have sometimes done harmful experiments on patients without their knowledge. (4) |  |  |  |  |
| Health care organizations don’t always keep your information totally private. (5) |  |  |  |  |
| Sometimes I wonder if health care organizations really know what they are doing. (6) |  |  |  |  |
| Mistakes are common in health care organizations. (7) |  |  |  |  |

Q84 How trustworthy are each of these actors which at various points have been involved in the development and distribution of the coronavirus vaccine?

|  | Completely (1) | Mostly (2) | Somewhat (3) | Not much (4) | Not at all (5) |
| --- | --- | --- | --- | --- | --- |
| Drug companies working to create and test the vaccine (1) |  |  |  |  |  |
| The U.S. Food and Drug Administration (FDA) (3) |  |  |  |  |  |
| The Trump/Pence Administration (4) |  |  |  |  |  |
| The Biden/Harris Administration (5) |  |  |  |  |  |
| Your usual doctor or healthcare team (Skip if you don’t have one) (6) |  |  |  |  |  |
| Pharmacies and walk-in clinics where people can get vaccinated (7) |  |  |  |  |  |
| Elected officials in your state (8) |  |  |  |  |  |
| Private agencies (i.e. businesses/start-ups) hired by government officials (9) |  |  |  |  |  |

Q93 How much do you agree or disagree with the following statements?

|  | Completely (1) | Mostly (2) | Somewhat (3) | Not Much (4) | Not at all (5) |
| --- | --- | --- | --- | --- | --- |
| Healthcare institutions provide the highest quality medical care. (1) |  |  |  |  |  |
| When treating my medical problems, health care institutions put my medical needs above all other considerations, including costs. (2) |  |  |  |  |  |
| Health care institutions will be held accountable if they cause me harm. (3) |  |  |  |  |  |
| Health care institutions treat all patients the same regardless of their race or ethnicity. (4) |  |  |  |  |  |
| Health care institutions only care about keeping medical costs down, and not what is needed for my health. (5) |  |  |  |  |  |

End of Block: Healthcare Mis/Trust Section

Start of Block: Governmental Mis/Trust Section

Q108 **Beyond medical care, we're interested in knowing how much you trust the federal government, your own state government, and your local government representatives.**

Q88 What percent of the time do you think you can trust the federal government in Washington to do what is best for the country?

|  | 0 | 10 | 20 | 30 | 40 | 50 | 60 | 70 | 80 | 90 | 100 |
| --- | --- | --- | --- | --- | --- | --- | --- | --- | --- | --- | --- |

| () | 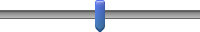 |
| --- | --- |

Q87 What percent of the time do you think you can trust your state government to make decisions in a fair way?

|  | 0 | 10 | 20 | 30 | 40 | 50 | 60 | 70 | 80 | 90 | 100 |
| --- | --- | --- | --- | --- | --- | --- | --- | --- | --- | --- | --- |

| () | 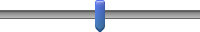 |
| --- | --- |

Q91 What percent of the time do you think you can trust your local government to make decisions in a fair way?

|  | 0 | 10 | 20 | 30 | 40 | 50 | 60 | 70 | 80 | 90 | 100 |
| --- | --- | --- | --- | --- | --- | --- | --- | --- | --- | --- | --- |

| () | 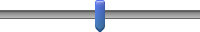 |
| --- | --- |

Display This Question:

If Please select the Race and/or Ethnicity that you most closely identify with? = American Indian/Native American/Alaska Native

Or Are you affiliated with a tribe(s)? = Yes (enter all tribal affiliations)

Q109 What percent of the time do you think you can trust your tribal government to make decisions in a fair way?

|  | 0 | 10 | 20 | 30 | 40 | 50 | 60 | 70 | 80 | 90 | 100 |
| --- | --- | --- | --- | --- | --- | --- | --- | --- | --- | --- | --- |

| () | 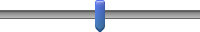 |
| --- | --- |

Display This Question:

If Please select the Race and/or Ethnicity that you most closely identify with? = American Indian/Native American/Alaska Native

Or Are you affiliated with a tribe(s)? = Yes (enter all tribal affiliations)

Q111 What percent of the time do you think you can trust your tribal government to do what is best for people in your area?

|  | 0 | 10 | 20 | 30 | 40 | 50 | 60 | 70 | 80 | 90 | 100 |
| --- | --- | --- | --- | --- | --- | --- | --- | --- | --- | --- | --- |

| () | 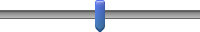 |
| --- | --- |

Display This Question:

If Please select the Race and/or Ethnicity that you most closely identify with? = American Indian/Native American/Alaska Native

Or Are you affiliated with a tribe(s)? = Yes (enter all tribal affiliations)

Q112 What percent of the time do you think you can trust the Indian Health Service to make decisions in a fair way?

|  | 0 | 10 | 20 | 30 | 40 | 50 | 60 | 70 | 80 | 90 | 100 |
| --- | --- | --- | --- | --- | --- | --- | --- | --- | --- | --- | --- |

| () | 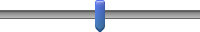 |
| --- | --- |

Display This Question:

If Please select the Race and/or Ethnicity that you most closely identify with? = American Indian/Native American/Alaska Native

Or Are you affiliated with a tribe(s)? = Yes (enter all tribal affiliations)

Q113 What percent of the time do you think you can trust the Indian Health Service to do what is best for the people in your area?

|  | 0 | 10 | 20 | 30 | 40 | 50 | 60 | 70 | 80 | 90 | 100 |
| --- | --- | --- | --- | --- | --- | --- | --- | --- | --- | --- | --- |

| () | 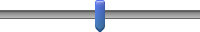 |
| --- | --- |

Q92 How often do you think the government in this country can be trusted to look out for the interests of your racial group?

- Always (1)
- Often (2)
- Occasionally (3)
- Rarely (4)
- Never (5)

End of Block: Governmental Mis/Trust Section

Start of Block: COVID-19 Impact Assessment Section

Q44 Between March 2020-March 2021, did you experience changes in any of the following?

|  | No (1) | Yes, and the change was positive (2) | Yes, and the change was negative (3) |
| --- | --- | --- | --- |
| Overall day-to-day life (1) |  |  |  |
| Housing (2) |  |  |  |
| Income (3) |  |  |  |
| Employment (4) |  |  |  |
| Financial Stress (5) |  |  |  |
| Health Services Use (6) |  |  |  |
| Depression (7) |  |  |  |
| Anxiety (8) |  |  |  |
| Stress (9) |  |  |  |
| Loneliness (10) |  |  |  |
| Emotional Support (11) |  |  |  |
| Racial Discrimination (12) |  |  |  |
| Vicarious Discrimination (13) |  |  |  |
| Amount of Sleep (14) |  |  |  |
| Quality of Sleep (15) |  |  |  |

Display This Question:

If Between March 2020-March 2021, did you experience changes in any of the following? != No

Q46 Do you believe the changes you experienced were due to the COVID-19 pandemic?

|  | Yes, change was due to the COVID-19 pandemic (1) |
| --- | --- |
| Overall day-to-day life (1) |  |
| Housing (2) |  |
| Income (3) |  |
| Employment (4) |  |
| Financial Stress (5) |  |
| Health Services Use (6) |  |
| Depression (7) |  |
| Anxiety (8) |  |
| Stress (9) |  |
| Loneliness (10) |  |
| Emotional Support (11) |  |
| Racial Discrimination (12) |  |
| Vicarious Discrimination (13) |  |
| Amount of Sleep (14) |  |
| Quality of Sleep (15) |  |

Q100 To what degree does your place of employment pose a risk of contracting COVID-19?

- No risk at all (1)
- A little risk (2)
- Some risk (3)
- A lot of risk (4)
- Extreme risk (5)

Q101 Do you personally know anyone that has been diagnosed with the coronavirus?

- Yes (1)
- No (2)

Q102 Do you personally know anyone in any of these categories?

|  | This has happened to me (1) | This has happened to someone that I’m close to (friend/family) (2) | This has happened to someone I know (acquaintance/colleague) (3) |
| --- | --- | --- | --- |
| Been exposed to someone with confirmed COVID-19 (1) |  |  |  |
| Been diagnosed with COVID-19 (2) |  |  |  |
| Been seriously ill with COVID-19 (3) |  |  |  |
| Been hospitalized with COVID-19 (4) |  |  |  |
| Has died from COVID-19 (5) |  |  |  |

End of Block: COVID-19 Impact Assessment Section

Start of Block: Access Section

Q123 A lot has happened in the last year due to the COVID-19 pandemic, we're interested in knowing whether you have a usual source of getting medical care, your level of access to medical care, and your experiences with that care.

Q122 Is there a particular doctor's office, clinic, health center, or another place that you usually go to if you are sick or need advice about your health?

- Yes (1)
- No (2)

Display This Question:

If Is there a particular doctor's office, clinic, health center, or another place that you usually g... = Yes

Q119 Where do you usually go if you are sick or need advice about your health?

- Doctor's office (1)
- Community health center (2)
- Hospital clinic or outpatient department (3)
- Hospital emergency room (4)
- Another place (5)

Display This Question:

If Is there a particular doctor's office, clinic, health center, or another place that you usually g... = Yes

Q124 On average how long does it usually take you to get to where you usually get health care from your home using your usual source of transportation (e.g., bus, train, car, etc.)?

- Less than 15 minutes (1)
- 16-30 minutes (2)
- 31-60 minutes (3)
- More than an hour (4)
- More than two hours (5)

Q125 In the last 12 months, was there any time when you needed medical care, but did not get it because you couldn’t afford it?

- Yes (1)
- No (2)

Q126 In the last 12 months, was there any time when you needed prescription drugs, but did not get it because you couldn’t afford it?

- Yes (1)
- No (2)

Q127 Do you have a doctor that you usually see if you are sick or need advice about your health?

- Yes (1)
- No (2)

Display This Question:

If Do you have a doctor that you usually see if you are sick or need advice about your health? = Yes

Q128 Please rate the frequency in which the following occurs:

|  | Never (1) | Sometimes (2) | Usually (3) | Always (4) |
| --- | --- | --- | --- | --- |
| My doctor explains things in a way that is easy to understand. (1) |  |  |  |  |
| My doctor shows respect for what I have to say. (2) |  |  |  |  |
| My doctor spends enough time with me. (3) |  |  |  |  |

Q129 During the last 2 years, did you need an interpreter to help you speak with a doctor?

- Yes (1)
- No (2)

Q132 In the last 12 months, have you had an appointment with a doctor, nurse, or other health professional by video or by phone?

- Yes (1)
- No (2)

Q133 In the last 12 months, has your usual source of care offered you an appointment with a doctor, nurse, or other health professional by video or by phone?

- Yes (1)
- No (2)

Q134 Before the coronavirus pandemic, did your usual source of care ever offer you an appointment with a doctor, nurse, or other health professional by video or by phone?

- Yes (1)
- No (2)

End of Block: Access Section

Start of Block: COVID-19 Perceived Risk Section

| 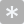 |
| --- |

Q143 Not including yourself, last year in 2020 how many other people lived in your household?

________________________________________________________________

Q52 Apart from yourself, how worried are you, if at all, about the possibility that your family members (living in your household) might catch the coronavirus?

- Very worried (1)
- Somewhat worried (2)
- Not very worried (3)
- Not worried at all (4)

Q53 Apart from yourself, how worried are you, if at all, about the possibility that your family members (not living in your household) might catch the coronavirus?

- Very worried (1)
- Somewhat worried (2)
- Not very worried (3)
- Not worried at all (4)

Q54 Apart from yourself, how worried are you, if at all, about the possibility that your close friends might catch the coronavirus?

- Very worried (1)
- Somewhat worried (2)
- Not very worried (3)
- Not worried at all (4)

Q55 Apart from yourself, how worried are you, if at all, about the possibility that some of your co-workers might catch the coronavirus?

- Very worried (1)
- Somewhat worried (2)
- Not very worried (3)
- Not worried at all (4)

End of Block: COVID-19 Perceived Risk Section

Start of Block: Governmental Mis/Trust - Police Violence Section

Q28 How much, if anything, have you read or heard of the circumstances of  Breonna Taylor’s death  in Louisville, Kentucky on March 13,  2020?

- A great deal (1)
- A lot (2)
- A moderate amount (3)
- A little (4)
- None at all (5)

Display This Question:

If How much, if anything, have you read or heard of the circumstances of Breonna Taylor’s death in L... != None at all

Q29 How satisfied are you with how Kentucky state and local government officials handled the investigation  into her death?

- Extremely satisfied (1)
- Somewhat satisfied (2)
- Neither satisfied nor dissatisfied (3)
- Somewhat dissatisfied (4)
- Extremely dissatisfied (5)

Q30 How much, if anything, have you read or heard of the circumstances of  George Floyd’s death  in Minneapolis, Minnesota on May 25,  2020?

- A great deal (1)
- A lot (2)
- A moderate amount (3)
- A little (4)
- None at all (5)

Display This Question:

If How much, if anything, have you read or heard of the circumstances of George Floyd’s death in Min... != None at all

Q31 How satisfied are you with how Minnesota state and local government officials handled the investigation into his death?

- Extremely satisfied (1)
- Somewhat satisfied (2)
- Neither satisfied nor dissatisfied (3)
- Somewhat dissatisfied (4)
- Extremely dissatisfied (5)

Q32 How much, if anything, have you read or heard of the circumstances of  Jacob Blakes’s shooting injury in Kenosha, Wisconsin on August 23,  2020?

- A great deal (1)
- A lot (2)
- A moderate amount (3)
- A little (4)
- None at all (5)

Display This Question:

If How much, if anything, have you read or heard of the circumstances of Jacob Blakes’s shooting inj... != None at all

Q33 How satisfied are you with how Wisconsin state and local government officials handled the investigation into his shooting?

- Extremely satisfied (1)
- Somewhat satisfied (2)
- Neither satisfied nor dissatisfied (3)
- Somewhat dissatisfied (4)
- Extremely dissatisfied (5)

Q94 How much, if anything, have you read or heard of the shooting by 17-year old Kyle Rittenhouse in Kenosha, Wisconsin on August 25,  2020?

- A great deal (1)
- A lot (2)
- A moderate amount (3)
- A little (4)
- None at all (5)

Display This Question:

If How much, if anything, have you read or heard of the shooting by 17-year old Kyle Rittenhouse in... != None at all

Q95 How satisfied are you with how Wisconsin state and local government officials handled the investigation into his shooting?

- Extremely satisfied (1)
- Somewhat satisfied (2)
- Neither satisfied nor dissatisfied (3)
- Somewhat dissatisfied (4)
- Extremely dissatisfied (5)

Q96 How much, if anything, have you read or heard of the circumstances of  Ernie Teddy Serrano’s death  in California on December 15, 2020?

- A great deal (1)
- A lot (2)
- A moderate amount (3)
- A little (4)
- None at all (5)

Display This Question:

If How much, if anything, have you read or heard of the circumstances of Ernie Teddy Serrano’s death... != None at all

Q97 How satisfied are you with how California state and local government officials handled the investigation into his death?

- Extremely satisfied (1)
- Somewhat satisfied (2)
- Neither satisfied nor dissatisfied (3)
- Somewhat dissatisfied (4)
- Extremely dissatisfied (5)

Q34 In general, how satisfied were you with how the Trump/Pence Administration responded to notable events of police violence in 2020?

- Extremely satisfied (1)
- Somewhat satisfied (2)
- Neither satisfied nor dissatisfied (3)
- Somewhat dissatisfied (4)
- Extremely dissatisfied (5)

Q35 In general, how satisfied were you with how the Biden/Harris Campaign responded to notable events of police violence in 2020?

- Extremely satisfied (1)
- Somewhat satisfied (2)
- Neither satisfied nor dissatisfied (3)
- Somewhat dissatisfied (4)
- Extremely dissatisfied (5)

Q36 In general, how confident do you feel that you will be satisfied by how the Biden/Harris Administration will sufficiently address incidents of police violence in 2021 and beyond?

- Extremely confident I will be satisfied (1)
- Very confident I will be satisfied (2)
- Moderately confident I will be satisfied (3)
- Slightly confident I will be satisfied (4)
- Not interesting at all (5)

| Page Break |  |
| --- | --- |

Q98 I, someone I’m close to, or someone I know have been… by the police (select all that apply)

|  | I/me (1) | Someone I'm related to (2) | Someone I'm close to (friend/colleague) (3) | Someone I know but am not close to (acquaintance) (4) | Someone that I know of but do not have a personal relationship with (5) |
| --- | --- | --- | --- | --- | --- |
| Stopped (1) |  |  |  |  |  |
| Arrested/Detained (2) |  |  |  |  |  |
| Disabled (someone who has a physical or mental impairment that substantially limits one or more major life activity after an encounter with the police) (3) |  |  |  |  |  |
| Framed (e.g., they planted drugs, etc.; police falsely alleged criminal involvements) (4) |  |  |  |  |  |
| Harassed (5) |  |  |  |  |  |
| Killed (6) |  |  |  |  |  |
| Physically abused or beaten (7) |  |  |  |  |  |
| Sexually assaulted or harassed (8) |  |  |  |  |  |
| Shot at (9) |  |  |  |  |  |
| Tased (10) |  |  |  |  |  |
| Threatened (11) |  |  |  |  |  |
| Verbally-assaulted or aggressed (12) |  |  |  |  |  |
| Other (13) |  |  |  |  |  |
| Other (14) |  |  |  |  |  |

End of Block: Governmental Mis/Trust - Police Violence Section

Start of Block: Race & Racism Section

Q37 We are all members of different social groups or social categories. We would like you to consider your race, ethnicity, or color in responding to the following statements. There are no right or wrong answers to any of these statements. We are interested in your honest reactions and opinions.

Q38 Please read each statement carefully, and respond by using the following scale from 1 to 5 with 1 as "Strongly Disagree" and 5 as "Strongly Agree".*

|  | Strongly agree (1) | Agree (2) | Neither agree nor disagree (3) | Disagree (4) | Strongly disagree (5) |
| --- | --- | --- | --- | --- | --- |
| Overall, being ${Q39/ChoiceGroup/SelectedChoices} has very little to do with how I feel about myself. (1) |  |  |  |  |  |
| I feel good about ${Q39/ChoiceGroup/SelectedChoices}s in the U.S. (2) |  |  |  |  |  |
| In general, being ${Q39/ChoiceGroup/SelectedChoices} is an important part of my self-image. (3) |  |  |  |  |  |
| I am happy that I am ${Q39/ChoiceGroup/SelectedChoices}. (4) |  |  |  |  |  |
| I feel that ${Q39/ChoiceGroup/SelectedChoices} peoples in the U.S. have made major accomplishments and advancements. (5) |  |  |  |  |  |
| My destiny is tied to the destiny of other ${Q39/ChoiceGroup/SelectedChoices}s in the U.S. (6) |  |  |  |  |  |
| Being ${Q39/ChoiceGroup/SelectedChoices} is unimportant to my sense of what kind of person I am. (7) |  |  |  |  |  |
| I have a strong sense of belonging to ${Q39/ChoiceGroup/SelectedChoices}s in the U.S. (8) |  |  |  |  |  |
| I often regret that I am ${Q39/ChoiceGroup/SelectedChoices}. (9) |  |  |  |  |  |
| I have a strong attachment to other ${Q39/ChoiceGroup/SelectedChoices}s in the U.S. (10) |  |  |  |  |  |
| Being ${Q39/ChoiceGroup/SelectedChoices} is an important reflection of who I am. (11) |  |  |  |  |  |
| Being ${Q39/ChoiceGroup/SelectedChoices} is not a major factor in my social relationships. (12) |  |  |  |  |  |
| In general, other Americans view ${Q39/ChoiceGroup/SelectedChoices}s in the US in a positive manner. (13) |  |  |  |  |  |
| I am proud to be ${Q39/ChoiceGroup/SelectedChoices}. (14) |  |  |  |  |  |
| I feel that ${Q39/ChoiceGroup/SelectedChoices} peoples in the U.S. have made valuable contributions to American society. (15) |  |  |  |  |  |

Q41 How often do you do the following things?

|  | Never (1) | About once a Month (2) | A few times a month (3) | About once a week (4) | A few times per week (5) | Almost every day (6) |
| --- | --- | --- | --- | --- | --- | --- |
| You try to prepare for possible insults from other people before leaving home. (1) |  |  |  |  |  |  |
| Feel that you always have to be very careful about your appearance (to get good service or avoid being harassed). (2) |  |  |  |  |  |  |
| Carefully watch what you say and how you say it. (3) |  |  |  |  |  |  |
| Try to avoid certain social situations in places. (4) |  |  |  |  |  |  |

Q115 Have you participated in Black Lives Matter rallies or protests against police brutality?

- Yes (1)
- No (2)

Q43 How many times in your life have you been discriminated against in each of the following ways, because of such things as your race, ethnicity, gender, age, religion, physical appearance, sexual orientation, or other characteristics?

(If the experience happened to you, but for some reason other than discrimination, enter "0".)

|  |  |
| --- | --- |
| You were not hired for a job or not given a promotion (1) | ▼ 0 (1) ... 11+ (11) |
| You were prevented from renting or buying a home in the neighborhood you wanted (2) | ▼ 0 (1) ... 11+ (11) |
| You were denied or provided inferior medical care (3) | ▼ 0 (1) ... 11+ (11) |
| You were denied or provided inferior service by a plumber, car mechanic, or other service provider (4) | ▼ 0 (1) ... 11+ (11) |

Q105 What was the main reason for the discrimination you experienced? (Select all that apply)

- Your age (1)
- Your gender (2)
- Your race (3)
- Your ethnicity or nationality (4)
- Your religion (5)
- Your height or weight (6)
- Some other aspect of your appearance (7)
- A physical disability (8)
- Your sexual orientation (9)
- Some other reason for discrimination (Please specify:) (10) ________________________________________________

End of Block: Race & Racism Section

Start of Block: Knowledge of Specific Governmental/Medical Human Rights Violations

Q56 How much, if anything, have you heard or read about the United States Federal Government’s Tuskegee Syphilis Study from 1932 to 1972, in which a group of Black men in Alabama who had syphilis were not told about it or treated for it?

- A great deal (1)
- A lot (2)
- A moderate amount (3)
- A little (4)
- None at all (5)

Q57 How much, if anything, have you heard or read about the United States Federal Government’s sterilization of Native American women and girls through the Indian Health Services in the mid-1960s to 1976?

- A great deal (1)
- A lot (2)
- A moderate amount (3)
- A little (4)
- None at all (5)

Q58 How much, if anything, have you heard or read about the United States Federal Government’s experiments in Guatemala, where prisoners, sex workers, and mentally ill patients were intentionally infected with Syphilis and Gonorrhea to test the effects of penicillin?

- A great deal (1)
- A lot (2)
- A moderate amount (3)
- A little (4)
- None at all (5)

Q141 How much, if anything, have you heard or read about the United States Federal Government’s detainment of immigrant children and families in unsanitary conditions and overcrowded facilities operated by the U.S. Immigration and Customs Enforcement (ICE)?

- A great deal (1)
- A lot (2)
- A moderate amount (3)
- A little (4)
- None at all (5)

End of Block: Knowledge of Specific Governmental/Medical Human Rights Violations

Start of Block: End of Survey Sociodemographics Section

Q61 Were you born in the United States?

- Yes (1)
- No (2)

Display This Question:

If Were you born in the United States? = No

Q62 In what year did you move to the United States?

▼ 1920 (1) ... 2021 (102)

Q63 In general, would you say your physical health is poor, fair, good, very good, or excellent?

- Excellent (5)
- Very good (1)
- Good (2)
- Fair (3)
- Poor (4)

Q103 In general, would you say your mental health is poor, fair, good, very good, or excellent?

- Excellent (5)
- Very good (1)
- Good (2)
- Fair (3)
- Poor (4)

Q64 Which best describes your current health insurance coverage?

- Insurance through an employer (my own or of a family member) (1)
- Medicaid (including Medicaid HMO/CMO) (2)
- Medicare (3)
- Military Health Care (Tricare) or VA (4)
- Indian Health Service (5)
- COBRA (6)
- Insurance purchased directly from an insurance company (including the ACA marketplace) (7)
- Uninsured (8)
- Other (9)

Q65 Thinking about all of your sources of income (e.g., jobs, unemployment insurance, stimulus payments), how much was your total family household income from all of these sources for the entire year in 2020?

▼ (1) ... $400,000 or more (11)

Q66 What is the highest level of education that you have completed?

- Less than 8th grade (1)
- Some High School, no diploma (2)
- High school diploma, GED, or equivalent (3)
- Some college, no degree (4)
- Associate's degree or another 2 year degree (5)
- Bachelor's Degree (6)
- Graduate Degree (i.e. MPH, MSW, MA) (7)
- Doctorate Degree (MD, PhD, EdD) (8)

Q67 What is your current employment status?

- Employed full time (1)
- Employed part time (2)
- Unemployed looking for work (3)
- Unemployed not looking for work (4)
- Retired (5)
- Student (6)
- Disabled (7)
- Temporarily Laid off or furloughed (8)
- Self-employed (9)

Q69 Do you currently have internet access in your home?

- Yes (1)
- No (2)

Q70 What is your current relationship status?

- Married or domestic partnership (1)
- Widowed (2)
- Divorced (3)
- Separated (4)
- Never married (5)

Q71 Generally speaking, do you consider yourself a Republican, Democrat, Independent, or something else?

- Republican (1)
- Democrat (2)
- Independent (3)
- Something else (4)

Q73 Which of these, if any, has been your primary source of news and information about the COIVD Pandemic?

- Social media (1)
- Broadcast news (ABC, CBS, NBC) (2)
- FOX News (3)
- CNN or MSNBC (4)
- Public television or radio (5)
- Newspaper/newspaper websites (6)
- Other news websites (7)
- Government websites (8)
- Family/friends (9)
- None of these (10)

End of Block: End of Survey Sociodemographics Section

Start of Block: End of Survey Block

Q76 Thank you for participating in our survey.

End of Block: End of Survey Block
